# Zero-Gap Tandem Photoelectrochemical Cell Based on Nanostructured WO_3_ and CuFe_2_O_4_ Photoelectrodes and a Proton-Exchange Membrane: Bias-Free Photoelectrochemical Operation and Degradation Analysis

**DOI:** 10.3390/nano16211415

**Published:** 2026-11-03

**Authors:** Rossana Giaquinta, Carmine Arnese, Stefano Trocino

**Affiliations:** Institute for Advanced Energy Technologies “Nicola Giordano” (ITAE), National Research Council of Italy (CNR), Via Salita S. Lucia Sopra Contesse 5, 98126 Messina, Italy; rossanagiaquinta@cnr.it (R.G.); carmine.arnese@cnr.it (C.A.)

**Keywords:** photoelectrochemical water splitting, tandem cell, zero-gap, tungsten oxide, copper ferrite, Nafion, bias-free, impedance spectroscopy, distribution of relaxation times, durability

## Abstract

Tandem photoelectrochemical (PEC) cells made exclusively of earth-abundant oxides are a promising route to solar hydrogen, but bias-free operation and durability remain challenging, particularly in acidic environments. This work presents a 1 cm^2^ zero-gap tandem PEC cell, operated without housing and fed only with deionized water, that integrates an electrodeposited nanostructured WO_3_ photoanode (band gap 2.75 eV), a spray-coated nanostructured CuFe_2_O_4_ photocathode (1.43 eV) and a Nafion 117 proton-exchange membrane acting as solid electrolyte and separator. Mott–Schottky analysis in N_2_-purged 0.1 M HClO_4_ confirms the expected n-type and p-type semiconductor character, with a band-edge alignment compatible with unassisted operation. Under simulated AM 1.5G sunlight, the device delivers ≈42 µA cm^−2^ at zero bias (solar-to-hydrogen efficiency ≈ 0.05%, assuming unity Faradaic efficiency) and a saturated photocurrent density of 0.47 mA cm^−2^ at −1.0 V (throughput efficiency 0.70%). After 96 h of operation (48 h at 0 V and 48 h at −0.7 V), the photocurrent decreases by only ≈5–6%. Impedance spectroscopy and distribution of relaxation times analysis identify a dominant millisecond-scale interfacial charge-transfer process. Post-operando analyses reveal that WO_3_ undergoes only mild surface reduction, whereas CuFe_2_O_4_ shows partial Cu^2+^ → Cu^+^ reduction and local delamination, identifying the photocathode as the lifetime-limiting component.

## 1. Introduction

Hydrogen produced from water and renewable energy is widely regarded as a key vector for the decarbonization of hard-to-abate sectors. At present, proton-exchange membrane [1,2] and anion-exchange membrane [3,4] water electrolyzers, together with solid oxide electrolysis cells [5], represent the most mature routes to green hydrogen; however, they rely on an external supply of renewable electricity and, in several cases, on critical raw materials. Among the emerging alternatives, photoelectrochemical (PEC) water splitting, first demonstrated by Fujishima and Honda on TiO_2_ [6], stands out because light harvesting, charge separation and electrocatalysis are integrated in a single device that converts sunlight directly into chemical energy stored in H_2_ [7,8,9]. Recent analyses of large-area devices and of their upscaling challenges indicate that the competitiveness of PEC hydrogen depends critically on inexpensive and abundant materials, on simple and scalable manufacturing routes and on device lifetimes well beyond the laboratory time scale [10,11].

In a PEC cell, photons absorbed by one or more semiconductor photoelectrodes create electron–hole pairs, which are used to drive the oxygen evolution reaction (OER) at the anode and the hydrogen evolution reaction (HER) at the cathode [12]. A single absorber must combine a band gap large enough to provide the photovoltage required for water splitting, i.e., well above the thermodynamic value of 1.23 eV once kinetic and ohmic losses are included, with band edges straddling the H^+^/H_2_ and O_2_/H_2_O redox levels; this combination strongly limits the fraction of the solar spectrum that can be harvested [7,13]. A promising solution is the tandem configuration [14], in which an n-type photoanode and a p-type photocathode with complementary band gaps are connected in series: the wide-band-gap absorber harvests the high-energy photons and drives the OER, whereas the narrow-band-gap absorber collects the transmitted light and drives the HER [15,16]. Bias-free (unassisted) operation becomes possible when the photocurrent–potential curves of the two photoelectrodes intersect at a non-zero current [14]. Since the pioneering work of Ohashi et al. [17], several all-oxide tandem configurations have been demonstrated, including p/n-Fe_2_O_3_ [18], TiO_2_/CaFe_2_O_4_ [19], BiVO_4_/Cu_2_O [20] and Fe_2_O_3_/CuBi_2_O_4_ [21], whereas solar-to-hydrogen (STH) efficiencies above 4% have been reached only with considerably more complex architectures [22].

Notably, most of these devices operate in neutral or alkaline electrolytes, which raise issues of buffering, carbonation and ohmic losses [23]. Acidic media are attractive in view of their high proton conductivity and of the possibility of coupling the photoelectrodes with proton-exchange membranes, but they are particularly demanding for the chemical stability of oxide photoelectrodes. In this context, Díaz-García and Gómez recently reported a WO_3_–CuCrO_2_ tandem cell that operated without bias in 0.1 M HClO_4_ for more than 3 h, showing that unprotected oxide photoelectrodes can split water in strongly acidic electrolytes [24].

Among n-type oxides, monoclinic WO_3_ is a particularly suitable photoanode for acidic environments, since it is chemically stable at low pH [25,26], shows good electron transport [27,28] and has a band gap of ≈2.6–2.8 eV, with a valence band lying well below the O_2_/H_2_O level that provides a large driving force for water oxidation [29,30,31]. Nevertheless, its photostability depends markedly on the electrolyte composition, because surface peroxo species and dissolution processes can develop during water oxidation [32]. On the photocathode side, the development of stable p-type oxides remains the main bottleneck: Cu_2_O and CuO are efficient absorbers but are prone to photocorrosion [33,34], and ternary oxides such as CuFeO_2_ [35], CaFe_2_O_4_ [36] and CuCrO_2_ [37] have therefore been investigated [38]. Copper ferrite (CuFe_2_O_4_) is a p-type spinel composed of abundant elements, with a narrow band gap that enables visible and near-infrared absorption and a photocurrent onset at rather positive potentials [39,40,41]. These features make CuFe_2_O_4_ a natural partner for WO_3_ in a tandem configuration: the photoanode absorbs the ultraviolet and blue part of the spectrum and transmits the remaining photons, which can be harvested by the underlying ferrite photocathode.

At the device level, our group has developed tandem photoelectrolysis cells in which a solid polymer electrolyte membrane is laminated between the photoanode and the photocathode in a zero-gap configuration. The membrane acts simultaneously as separator and ionic conductor, minimizes ohmic losses, avoids the handling of corrosive liquid electrolytes and enables the production of dry, separated gases [42]. This concept has been progressively scaled up to 10 cm^2^ [43] and 25 cm^2^ [44] devices based on critical raw material-free hematite and cupric oxide photoelectrodes and anion-exchange membranes, and the role of earth-abundant co-catalysts has been systematically investigated [45,46]. In these studies, electrochemical impedance spectroscopy (EIS) and the distribution of relaxation times (DRT) proved to be powerful tools to deconvolute ohmic, transport and interfacial contributions in membrane-based devices [47,48,49], and post-operando analyses identified the CuO photocathode, progressively reduced under operation, as the lifetime-limiting component of our 10 cm^2^ prototype. Proton-exchange membrane PEC cells have also been demonstrated with WO_3_ photoanodes coupled with Pt-based dark cathodes, e.g., for the splitting of water vapor [50,51]. Despite this progress, a zero-gap tandem cell in which an acid-stable WO_3_ photoanode is coupled with a CuFe_2_O_4_ photocathode through a proton-exchange membrane has not yet been reported, and the durability of such an all-oxide device in contact with a strongly acidic ionomer is unknown. In addition, studies linking the evolution of the device performance to specific structural, chemical and morphological changes in the photoelectrodes remain scarce.

Building on these premises, in the present work we design and characterize a 1 cm^2^ zero-gap tandem cell in which a Nafion 117 proton-exchange membrane is sandwiched between an electrodeposited nanostructured WO_3_ photoanode and a spray-coated nanostructured CuFe_2_O_4_ photocathode, both supported on fluorine-doped tin oxide (FTO) glass. The cell is operated without any external housing and fed only with deionized water, so that the membrane provides at the same time the acidic environment, the ionic path and the separation of the anodic and cathodic products. Within this framework, the explicit questions guiding the study are:

(i) Can a zero-gap tandem cell based on nanostructured WO_3_ and CuFe_2_O_4_ photoelectrodes and a proton-exchange membrane operate without external bias, and how does its response evolve under a moderate assisting bias?
(ii) How do the optical and electronic properties of the two photoelectrodes and the interfacial charge-transfer processes, as resolved by EIS and DRT, determine the device performance?
(iii) Which structural, chemical and morphological changes occur in the photoelectrodes after 96 h of operation, as revealed by post-operando analyses, and which component limits the lifetime of the device?

## 2. Materials and Methods

This section describes the materials, the preparation of the photoelectrodes, the assembly of the zero-gap tandem cell and the characterization techniques.

### 2.1. Materials

The tungsten precursor for the WO_3_ photoanode was sodium tungstate dihydrate (Na_2_WO_4_·2H_2_O), while the CuFe_2_O_4_ photocathode was prepared from copper(II) nitrate trihydrate (Cu(NO_3_)_2_·3H_2_O) and iron(III) nitrate nonahydrate (Fe(NO_3_)_3_·9H_2_O). Hydrogen peroxide (H_2_O_2_, 40%), nitric acid (HNO_3_), absolute ethanol, isopropyl alcohol and perchloric acid (HClO_4_) were also employed. All chemicals were of analytical grade (Sigma-Aldrich, St. Louis, MO, USA) and were used as received. Both photoelectrodes were deposited on FTO-coated glass (Sigma-Aldrich). The solid polymer electrolyte was a commercial Nafion™ 117 proton-exchange membrane (nominal thickness 183 µm; Chemours, Wilmington, DE, USA), used as received without any pretreatment. Deionized water was used for all solutions and for feeding the cell.

### 2.2. Preparation of the WO_3_ Photoanode

The FTO substrate was first cleaned by sonication in ethanol and in distilled water (10 min each) and dried at room temperature. WO_3_ films were then grown by cathodic electrodeposition from an acidic peroxotungstate bath, following procedures previously reported in the literature [52,53]. In particular, the bath was prepared by dissolving 4.12 g of Na_2_WO_4_·2H_2_O (25 mM) in deionized water and adding 11.3 mL of 40% H_2_O_2_, which produced a yellow peroxotungstate solution; HNO_3_ was then added until pH 1.2 was reached, and the final volume was adjusted to 500 mL. Electrodeposition was performed with an Autolab PGSTAT302N potentiostat/galvanostat (Metrohm Autolab, Utrecht, The Netherlands) in a conventional three-electrode configuration, using the cleaned FTO glass as working electrode, a platinum wire as counter electrode and an Ag/AgCl reference electrode (both from AMEL, Milan, Italy), placed at equal distances in the bath. A constant potential of −0.27 V vs. Ag/AgCl was applied for 40 min (1 cm^2^ geometric area), in the dark and under gentle, constant stirring. The resulting films were rinsed thoroughly with doubly distilled water to remove residual ions, dried at room temperature and calcined in air at 450 °C for 1 h to convert the deposit into crystalline WO_3_.

### 2.3. Preparation of the CuFe_2_O_4_ Photocathode

CuFe_2_O_4_ films were deposited by spray coating on FTO glass cleaned as described above. The precursor solution was obtained by dissolving 4.61 mg of Cu(NO_3_)_2_·3H_2_O and 15.39 mg of Fe(NO_3_)_3_·9H_2_O (Cu:Fe molar ratio = 1:2) in a mixture of 1.8 mL of absolute ethanol and 0.2 mL of deionized water under magnetic stirring. A volume of 1 mL of this solution was sprayed onto the substrate (1 cm^2^ geometric area), kept at 60 °C, using a dual-action airbrush (Custom Micron CM-C2 Plus; ANEST IWATA Corporation, Yokohama, Japan) with compressed air at 2 bar as carrier gas and a nozzle-to-substrate distance of 0.5 cm, without intermediate drying steps. A final annealing at 600 °C for 2 h in air (heating rate 2 °C min^−1^) completed the transformation of the nitrate precursors into the CuFe_2_O_4_ spinel phase.

### 2.4. Assembly of the Zero-Gap Tandem Cell

**Figure 1:**
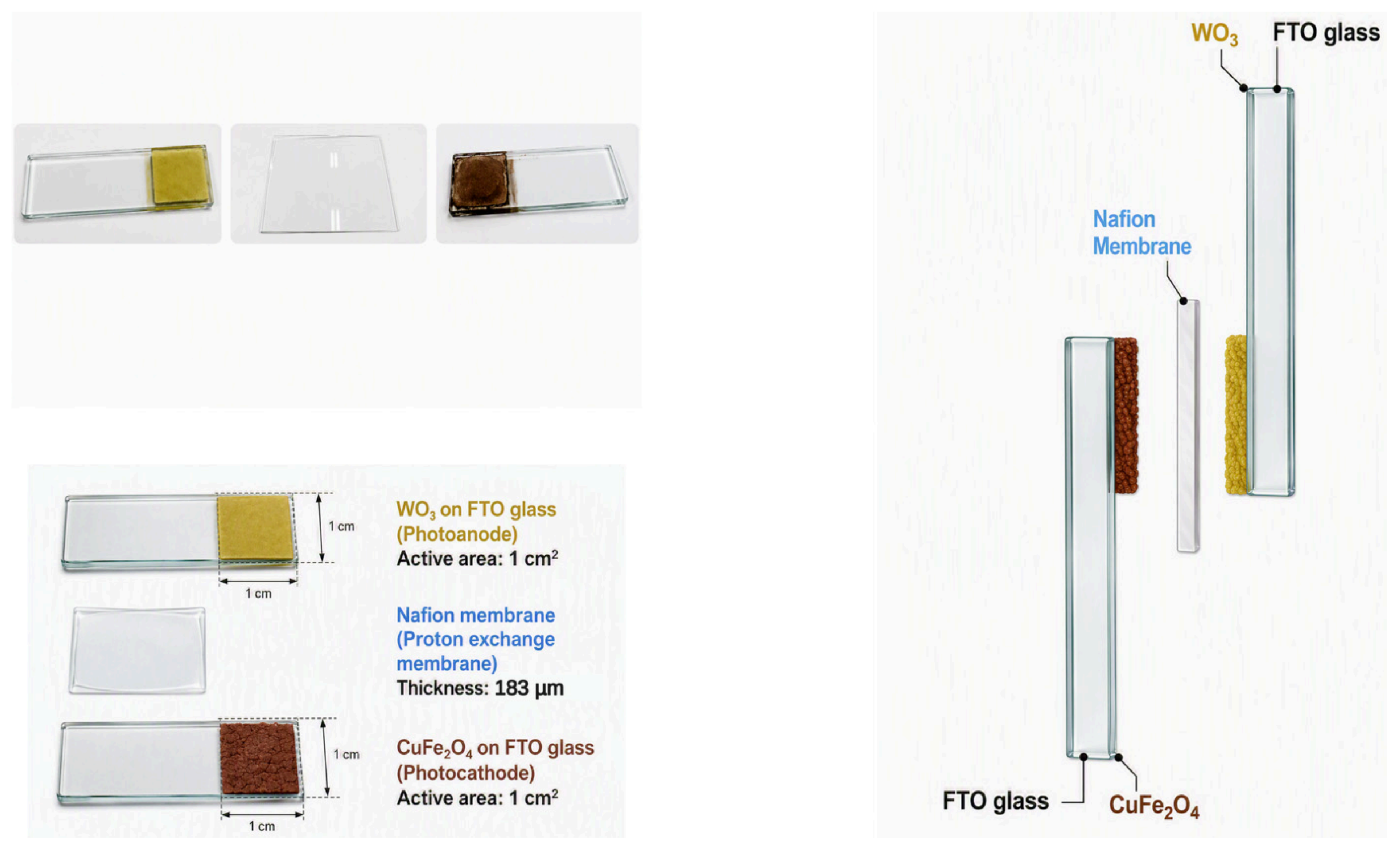
Components and assembly of the zero-gap tandem photoelectrochemical cell. Top left: photographs of the WO_3_/FTO photoanode, the Nafion 117 membrane and the CuFe_2_O_4_/FTO photocathode. Bottom left: sketch of the components with the active electrode areas (1 cm^2^). Right: schematic side view of the assembled cell, with both oxide layers facing the membrane. Illumination is provided through the FTO glass of the WO_3_ photoanode.

The cell relies on a zero-gap architecture, in which a solid proton-exchange membrane acts both as separator and as ionic conductor, as in our previous tandem devices [42]. The components and the assembly of the cell are shown in Figure 1. The WO_3_ photoanode appears as a pale yellow film and the CuFe_2_O_4_ photocathode as a reddish-brown film, both with an active area of 1 cm^2^. The cell was built by superimposing the WO_3_/FTO photoanode, the Nafion 117 membrane and the CuFe_2_O_4_/FTO photocathode, with both oxide layers facing the membrane and the two active areas aligned, so that the membrane provided the only ionic path between the electrodes. No external housing was used. The membrane, larger than the electrode overlap, was wetted with deionized water by means of a micropipette at its edges protruding outside the cell, and water uptake by the ionomer kept the active region hydrated during operation. Electrical contacts were made on the uncoated FTO regions of the two electrodes. During all measurements, the cell was kept under a continuous N_2_ flow surrounding the cell; therefore, atmospheric O_2_ was excluded, and the only O_2_ and H_2_ present were those produced by the device.

During operation, the n-type WO_3_ photoanode, illuminated through its FTO glass, absorbs the high-energy part of the incident spectrum; the photogenerated holes oxidize water to O_2_ at the WO_3_ surface, releasing protons, while the electrons are collected by the FTO and reach the photocathode through the external circuit. Since only the photons that are not absorbed by WO_3_ cross the membrane, the p-type CuFe_2_O_4_ layer is excited mainly by visible light of longer wavelength, and its photogenerated electrons reduce protons to H_2_. Charge neutrality is preserved by the migration of protons through the Nafion 117 membrane, which also acts as a physical barrier between the evolved gases.

### 2.5. Photoelectrochemical Characterization

All photoelectrochemical measurements were performed with an Autolab PGSTAT302N potentiostat/galvanostat equipped with a frequency response analyzer (FRA) module (Metrohm Autolab), using a solar simulator (Oriel Sol3A Class AAA, Newport, Irvine, CA, USA) calibrated to AM 1.5G (100 mW cm^−2^) with a calibrated reference cell (91150V, Newport). The tandem cell was measured in a two-electrode configuration, the cell voltage (*V*) being applied between the photoanode and the photocathode. Current density–voltage (*J*–*V*) curves were recorded at 20 mV s^−1^ from short circuit (0 V) to −1.3 V in the dark, under continuous illumination and under chopped illumination (5 s light/5 s dark). Data are reported using the photodiode convention, i.e., the hydrogen-evolution current is plotted as negative and an assisting (reverse) cell bias is reported as a negative voltage. The photocurrent density was calculated as *j*_p_ = *j*_light_ − *j*_dark_. No smoothing, filtering or baseline correction was applied to the *J*–*V* and chronoamperometric data, which were only normalized to the 1 cm^2^ geometric area; *j*_p_ and the derived efficiencies were calculated point by point from the continuous-light and dark curves. For graphical representation, consecutive experimental points of the *J*–*V* and chronoamperometric curves were connected using the smoothed-line option of the plotting software (Microsoft Excel for Microsoft 365; Microsoft Corporation, Redmond, WA, USA); this option affects only the display between adjacent data points, and all values reported in the text and tables were obtained from the original data.

The performance of the device under bias was evaluated in terms of throughput efficiency (*η*_tp_), quantified by [13]:

(1) $$\eta_{\mathrm{tp}}(\%)=\frac{\left|j_{p}\right|\times E_{\mathrm{tn}}}{P_{\mathrm{in}}+\left|j_{p}\times V_{\mathrm{bias}}\right|}\times 100$$

In this expression, *E*_tn_ = 1.48 V is the thermoneutral voltage of liquid water splitting, *V*_bias_ is the applied cell voltage and *P*_in_ = 100 mW cm^−2^ is the incident irradiance. Because *E*_tn_ includes both the Gibbs free-energy and the entropic contributions, *η*_tp_ expresses, on a higher-heating-value basis, the share of the overall (optical plus electrical) input power that is converted into chemical energy of hydrogen. At zero bias, the solar-to-hydrogen efficiency was calculated as [13,54]:

(2) $$\mathrm{STH}(\%)=\frac{\left|j_{\mathrm{sc}}\right|\times 1.23\;V\times \eta_{F}}{P_{\mathrm{in}}}\times 100$$

where *j*_sc_ is the current density at short circuit (0 V) under illumination and *η*_F_ is the Faradaic efficiency for H_2_ evolution, assumed to be unity.

Device stability was assessed by a 96 h potentiostatic protocol under continuous AM 1.5G illumination, consisting of 48 h at 0 V (bias-free operation) followed by 48 h at −0.7 V, with 30 min dark intervals every 12 h as internal reference conditions. *J*–*V* curves and impedance spectra were recorded on the fresh cell at the beginning of test (BoT) and at the end of the protocol (end of test, EoT) under identical conditions.

### 2.6. Electrochemical Impedance Spectroscopy and Distribution of Relaxation Times

EIS spectra were recorded in potentiostatic mode at cell voltages of 0, −0.7 and −1.3 V, in the dark and under AM 1.5G illumination, over the 100 kHz–10 mHz frequency range with a single-sine excitation of 10 mV_rms_, at both BoT and EoT. The spectra were processed with the NOVA 2.1.7 software (Metrohm Autolab) and fitted with an *R*_s_(*R*_p1_‖CPE_1_)(*R*_p2_‖CPE_2_) equivalent circuit, in which *R*_s_ is the series (ohmic) resistance and each resistance *R*_p_ in parallel with a constant phase element (CPE) describes a relaxation process. The impedance of each CPE and the characteristic time constant of each *R*‖CPE element were calculated as:

(3) $$Z_{\mathrm{CPE}}=\frac{1}{Y{\left(\mathrm{j\omega}\right)}^{n}}$$

(4) $$\tau ={\left(R\cdot Y\right)}^{\frac{1}{n}}$$

where *Y* and *n* are the CPE parameters, *ω* is the angular frequency and *j* is the imaginary unit.

Selected spectra were also analyzed with the DRT method [48], which describes the impedance as:

(5) Z(ω)=Rs+∫−∞+∞γ(τ)1+jωτ dlnτ

where *γ*(*τ*) is the distribution function of the relaxation times. The function *γ*(*τ*) was obtained by Tikhonov regularization (ridge, *λ* = 1.0) [55,56,57], implemented in an in-house script written in Python 3.15 (Python Software Foundation, Beaverton, OR, USA), using a logarithmically spaced grid of relaxation times between 10^−8^ and 10^1^ s; the regularization penalizes first-order differences in *γ*(*τ*), thus suppressing artificial oscillations, while leaving *R*_s_ unpenalized. The local maxima of *γ*(*τ*) were identified as the characteristic processes, and their amplitude and central relaxation time were compared between BoT and EoT. The aims of this analysis were (i) to separate the ohmic contribution from the interfacial processes without assuming an equivalent circuit, (ii) to identify the dominant relaxation times and their intensity, and (iii) to compare quantitatively the fresh and the aged device.

### 2.7. Mott–Schottky Measurements

Mott–Schottky measurements were carried out on individual WO_3_ and CuFe_2_O_4_ electrodes in a conventional three-electrode half-cell, with an Ag/AgCl (saturated KCl) reference electrode and a platinum wire counter electrode (both from AMEL), in N_2_-purged 0.1 M HClO_4_, using the Autolab PGSTAT302N potentiostat/galvanostat equipped with the FRA module. The space-charge capacitance (*C*) was measured in the dark at a fixed frequency of 1 kHz as a function of the applied potential (*E*). The conductivity type and the flat-band potential were derived from the sign of the slope and from the potential-axis intercept of the linear region of the 1/*C*^2^ vs. *E* plots, according to the Mott–Schottky relation [58]:

(6) $$\frac{1}{C^{2}}=\pm \frac{2}{{e\varepsilon \varepsilon }_{0}N}\left(E-E_{\mathrm{fb}}-\frac{k_{B}T}{e}\right)$$

where *e* is the elementary charge, *ε* and *ε*_0_ are the relative permittivity of the semiconductor and the vacuum permittivity, *N* is the majority-carrier density, *k*_B_ is the Boltzmann constant and *T* is the absolute temperature; the positive and negative signs apply to n-type and p-type semiconductors, respectively. The potentials of the Mott–Schottky measurements, measured vs. the Ag/AgCl reference electrode in the three-electrode configuration, were converted to the reversible hydrogen electrode (RHE) scale according to the Nernst equation:

*E*_RHE_ = *E*_Ag/AgCl_ + 0.197 V + 0.059 V × pH (7)

where 0.197 V is the potential of the Ag/AgCl (saturated KCl) reference electrode vs. the standard hydrogen electrode at 25 °C and 0.059 V corresponds to 2.303*RT*/*F* at 25 °C; for 0.1 M HClO_4_ (pH 1), *E*_RHE_ = *E*_Ag/AgCl_ + 0.256 V. All Mott–Schottky data are therefore reported vs. RHE (Figure S1c,d).

The acidic electrolyte was deliberately selected to reproduce the strongly acidic environment experienced by both photoelectrodes in contact with the sulfonic acid groups of the Nafion ionomer in the zero-gap cell, so that the flat-band potentials could be used directly to construct the energy-level diagram of the device (Section 3.2). Moreover, the potential region used for the analysis corresponds to depletion conditions, in which the concentration of minority carriers in the dark, i.e., of electrons in p-type CuFe_2_O_4_ and of holes in n-type WO_3_, is negligible; under these conditions, the electrodes behave as nearly blocking interfaces, and faradaic processes, including the electrochemical self-reduction of Cu^2+^, are strongly suppressed. To verify the stability of CuFe_2_O_4_ in this electrolyte, the Mott–Schottky measurements on the photocathode in N_2_-purged 0.1 M HClO_4_ were repeated several times on the same electrode; the Mott–Schottky plots and the resulting flat-band potentials were reproducible, indicating that CuFe_2_O_4_ is stable in this acidic solution under the measurement conditions.

### 2.8. UV–Vis Spectroscopy and Optical Analysis

UV–Vis absorbance spectra of the individual cell components (WO_3_ on FTO, Nafion 117 membrane and CuFe_2_O_4_ on FTO) were recorded in the 300–900 nm range with a Cary 6000i UV–Vis–NIR spectrophotometer (Agilent Technologies, Santa Clara, CA, USA). The fraction of incident light transmitted by each layer was derived from the measured absorbance (*A*) using the Beer–Lambert law, and the spectral irradiance reaching the CuFe_2_O_4_ photocathode in the assembled tandem cell was estimated by cascading the transmittances of the overlying layers:

(8) $$T_{i}\left(\lambda \right)=10^{-A_{i}\left(\lambda \right)}$$

*F*_CFO_(*λ*) = *T*_WO_3__(*λ*) *T*_N_(*λ*) [1 − *T*_CFO_(*λ*)] (9)

*F*_tot_(*λ*) = [1 − *T*_WO_3__(*λ*)] + *F*_CFO_(*λ*) (10)

where *F*_CFO_(*λ*) is the fraction of the incident light absorbed by CuFe_2_O_4_, *F*_tot_(*λ*) is the combined photoactive capture of the two photoelectrodes and the subscripts WO_3_, N and CFO refer to the WO_3_ photoanode, the Nafion 117 membrane and the CuFe_2_O_4_ photocathode, respectively. The fraction of the WO_3_-transmitted light captured by CuFe_2_O_4_ was calculated as *F*_CFO_(*λ*)/*T*_WO3_(*λ*). Losses due to reflection and scattering were neglected. The optical band gaps were determined by the Tauc method for direct allowed transitions [59,60,61]:

(*Ahν*)^2^ = *B*(*hν* − *E*_g_) (11)

where *A* is taken as proportional to the absorption coefficient, *hν* is the photon energy and *B* is a constant; *E*_g_ was obtained by extrapolating the linear portion of the absorption edge to the photon-energy axis.

### 2.9. Structural, Morphological and Surface Characterization

X-ray diffraction (XRD), field-emission scanning electron microscopy (FE-SEM) coupled with energy-dispersive X-ray spectroscopy (EDS), transmission electron microscopy (TEM) and X-ray photoelectron spectroscopy (XPS) measurements were performed first on “fresh” electrodes (as prepared, before any electrochemical measurement) and then on “used” electrodes recovered from the tandem cell after the EoT characterization.

Crystallographic analysis was carried out directly on the electrodes supported on FTO glass with a Bruker D8 Advance diffractometer (Bruker AXS, Karlsruhe, Germany), using Ni-filtered Cu Kα radiation (*λ* = 1.5406 Å) at 40 kV and 40 mA, with an angular step of 0.03°. Phases were identified by comparison with the reference patterns of the Powder Diffraction File (PDF; International Centre for Diffraction Data, Newtown Square, PA, USA) for monoclinic WO_3_ (PDF 43-1035), tetragonal spinel CuFe_2_O_4_ (PDF 34-0425) and tetragonal SnO_2_ (PDF 72-1147).

The morphology and the elemental composition of the films were examined with a Thermo Scientific Helios 5 UC DualBeam FE-SEM (Thermo Fisher Scientific, Waltham, MA, USA) operated in high-resolution imaging mode and equipped with a Thermo Scientific UltraDry silicon drift detector (SDD) for EDS; EDS spectra were acquired and processed with the Thermo Scientific Pathfinder X-ray Microanalysis Software. Fresh and used electrodes were examined under identical imaging conditions, to enable a direct comparison of texture, grain size and possible operation-induced changes. TEM analysis was performed with a JEOL JEM-F200 microscope (JEOL Ltd., Tokyo, Japan) on material removed from the electrodes, ultrasonically dispersed in isopropyl alcohol and deposited on holey carbon-coated Cu grids (Agar Scientific, Stansted, UK).

XPS was employed to investigate the changes in surface composition and oxidation states of both photoelectrodes after operation. Measurements were carried out with a Physical Electronics PHI 5800-01 spectrometer (Physical Electronics, Chanhassen, MN, USA) equipped with a monochromatic Al Kα X-ray source. The binding-energy scale was referenced to the adventitious C 1s C–C/C–H component at 284.8 eV. High-resolution regions were processed with the MultiPak software (Physical Electronics) and an in-house Python 3.15 script, using a Shirley background and mixed Gaussian–Lorentzian line shapes. For the W 4f region, the W 4f_7/2_–4f_5/2_ separation and the doublet area ratio were constrained to 2.17 eV and 4:3, respectively, and the spectral window was extended to include the W 5p_3/2_ contribution near 41.6 eV. The Cu 2p region was described by the Cu^2+^ multiplet envelope and its shake-up satellites, and the Cu LMM Auger region was used to discriminate Cu^+^ from Cu^0^ [62]; the Fe 2p region was described by an Fe^3+^ multiplet model including both satellites near 719 and 733 eV [63,64]. For the used WO_3_, the C 1s, F 1s and S 2p regions were also acquired to verify the presence of residual ionomer [65].

### 2.10. Use of Generative Artificial Intelligence

Generative artificial intelligence tools were used to assist in structuring the manuscript, drafting and editing the text, formatting it according to the journal template and assembling multi-panel figures from the original graphs produced by the authors. No experimental data were generated or modified by these tools; all data, analyses and interpretations were produced and verified by the authors.

## 3. Results and Discussion

### 3.1. Optical Properties and Spectral Complementarity

**Figure 2:**
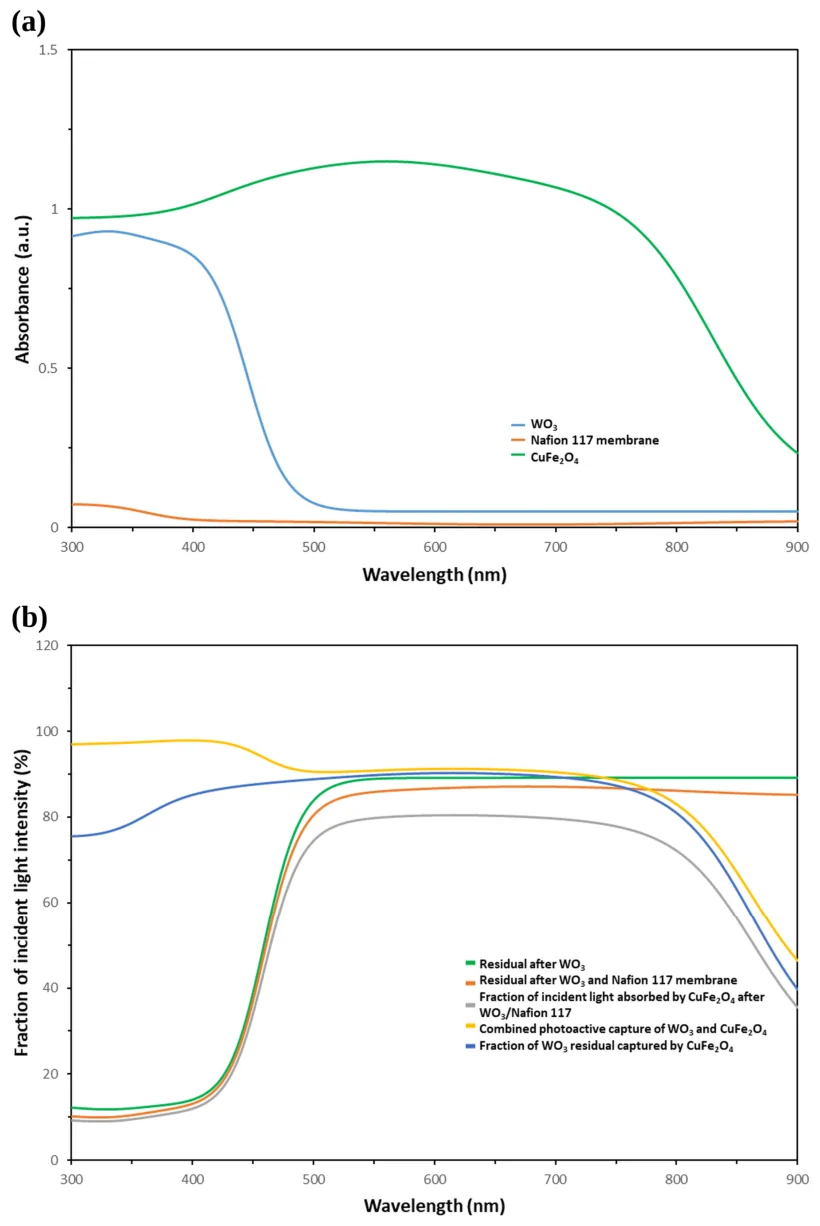
(**a**) UV–Vis absorbance spectra of the WO_3_/FTO photoanode, the Nafion 117 membrane and the CuFe_2_O_4_/FTO photocathode in the 300–900 nm range. (**b**) Spectral distribution of the incident light intensity in the assembled tandem cell, calculated from the absorbance spectra with the Beer–Lambert law: fraction transmitted by WO_3_, fraction transmitted by the WO_3_/Nafion 117 assembly, fraction of the incident light absorbed by CuFe_2_O_4_, combined photoactive capture of WO_3_ and CuFe_2_O_4_, and fraction of the WO_3_-transmitted light captured by CuFe_2_O_4_.

Figure 2a reports the UV–Vis absorbance spectra of the individual components of the tandem cell. WO_3_ exhibits a steep absorption edge at ≈450–470 nm, with an absorbance close to 0.9 below 420 nm and of only ≈0.05 above 520 nm; the photoanode is therefore essentially transparent to visible light of longer wavelength. In contrast, CuFe_2_O_4_ absorbs strongly across the whole visible range, with an absorbance above 1 between ≈400 and ≈760 nm and a maximum of ≈1.15 at ≈560 nm, and its absorbance decreases only beyond ≈780 nm, reaching ≈0.23 at 900 nm. The Nafion 117 membrane is highly transparent, with an absorbance lower than 0.08 at 300 nm and of ≈0.02 above 400 nm.

In this context, the optical budget of the assembled tandem cell was quantified from the absorbance spectra by means of the Beer–Lambert law (Section 2.8), as shown in Figure 2b. The WO_3_ photoanode acts as a long-pass optical filter: it transmits only ≈12% of the incident light below 420 nm, but ≈89% above 520 nm, while the membrane causes only a minor additional attenuation, so that ≈87% of the incident light above 520 nm reaches the photocathode. The CuFe_2_O_4_ layer absorbs ≈80% of the incident photons between ≈520 and 700 nm, i.e., ≈90% of the light transmitted by WO_3_, and still ≈36% at 900 nm. As a result, the combined photoactive capture of the two photoelectrodes is ≈97% between 300 and 420 nm and ≈90–91% between 500 and 720 nm, exceeding 90% over most of the 300–740 nm range. Since reflection and scattering were neglected, these values should be regarded as upper limits; nevertheless, they validate the spectral complementarity of the WO_3_/CuFe_2_O_4_ pair, in which the photoanode harvests the photons with *λ* < ≈460 nm and the photocathode collects most of the transmitted visible light, which is the basic requirement of the spectral-splitting tandem concept [14].

### 3.2. Band Gaps, Flat-Band Potentials and Energy-Level Alignment

Tauc analysis of the absorbance data (Figure S1a,b) yields optical band gaps of 2.75 eV for WO_3_ and 1.43 eV for CuFe_2_O_4_, corresponding to absorption onsets at ≈451 and ≈867 nm, respectively [59,60]. The value obtained for WO_3_ falls within the 2.6–2.8 eV range commonly reported for monoclinic WO_3_ films [24,29], whereas the narrow band gap of CuFe_2_O_4_ agrees with the visible–near-infrared absorption reported for copper ferrite photocathodes [39,40,41] and with the spectral distribution of Figure 2, confirming the spectral complementarity of the tandem architecture.

Furthermore, the Mott–Schottky plots recorded in N_2_-purged 0.1 M HClO_4_ (Figure S1c,d) provide experimental confirmation of the conductivity type of both photoelectrodes. For WO_3_, the 1/*C*^2^ vs. potential plot shows a positive slope, indicative of n-type conductivity, with a flat-band potential (*E*_fb_) of +0.38 V vs. RHE (+0.12 V vs. Ag/AgCl); the deviation from linearity at low potentials can be ascribed to the onset of accumulation conditions and to the contribution of surface states [58]. Conversely, the negative slope observed for CuFe_2_O_4_ confirms p-type conductivity (the conductivity type of copper ferrite is known to depend on its defect chemistry and preparation conditions [39,66]), with *E*_fb_ = +0.82 V vs. RHE (+0.56 V vs. Ag/AgCl). The potentials measured vs. Ag/AgCl were converted to the RHE scale with the Nernst equation (pH 1, Equation (7)). Although flat-band potentials determined at a single frequency are approximate [58], the sign of the slopes unambiguously identifies the expected n-type and p-type character of the photoanode and of the photocathode. Since the surface of oxide semiconductors is in protonation equilibrium with the electrolyte, their flat-band potentials typically shift by ≈−59 mV per pH unit, so that the band-edge positions on the RHE scale, which are those relevant for water splitting, are expected to be largely independent of pH [12,58]; the values measured at pH 1 are therefore representative of the acidic environment provided by the ionomer in the device. The Mott–Schottky measurements on CuFe_2_O_4_, repeated several times in N_2_-purged 0.1 M HClO_4_, gave reproducible plots and flat-band potentials, confirming that the photocathode is stable in this electrolyte under the measurement conditions (Section 2.7). Consistently, the post-operando analyses (Section 3.8, Section 3.9 and Section 3.10) show that even 96 h of operation in contact with the acidic ionomer did not alter the bulk phase or the Cu/Fe ratio of the photocathode.

**Figure 3:**
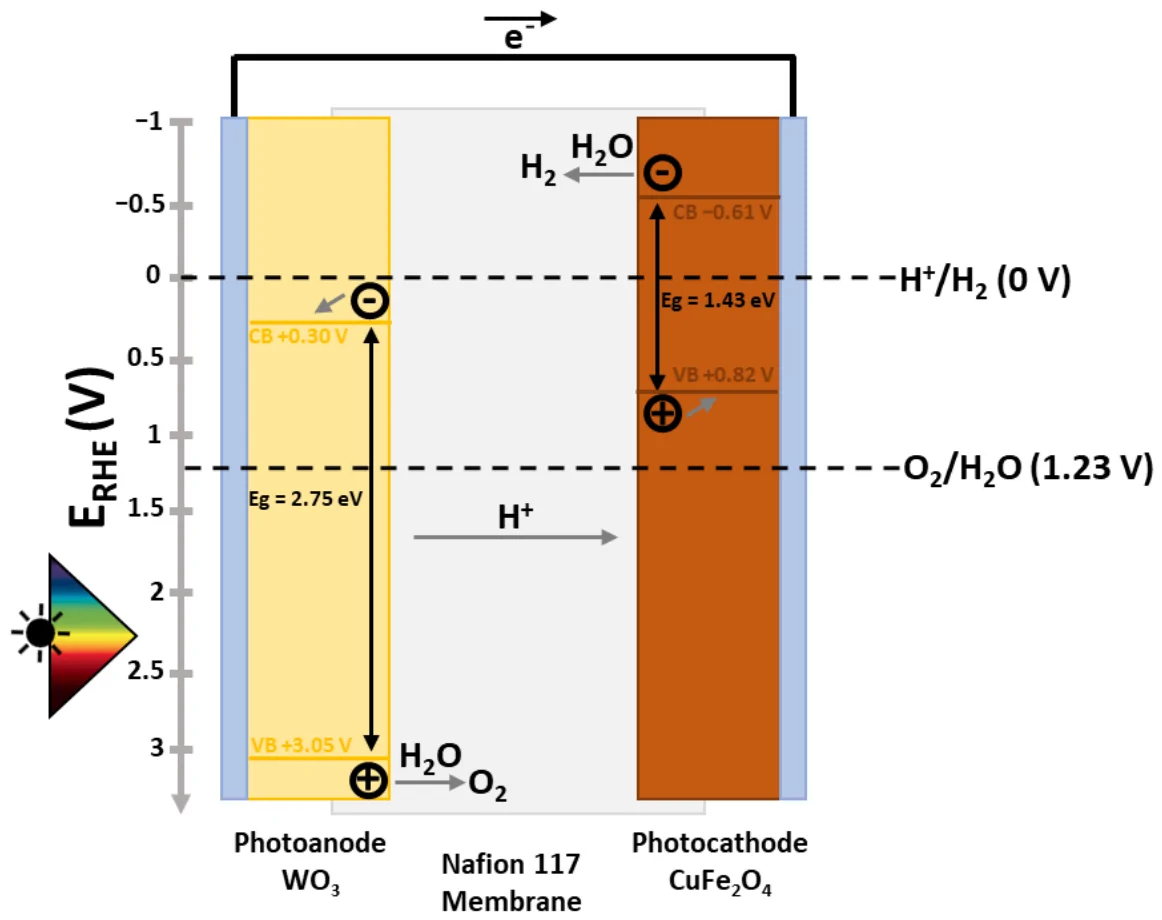
Schematic operating principle and energy-level diagram of the zero-gap WO_3_/Nafion 117/CuFe_2_O_4_ tandem photoelectrochemical cell. Band-edge positions vs. RHE were derived from the optical band gaps and the flat-band potentials (Figure S1). Under illumination, photogenerated holes in WO_3_ oxidize water to O_2_, photogenerated electrons in CuFe_2_O_4_ reduce protons to H_2_, majority carriers recombine through the external circuit, and H^+^ ions migrate through the Nafion 117 membrane. Circled minus and plus signs denote photogenerated electrons and holes, respectively; grey arrows indicate their transfer to the back contacts and to the reactions at the photoelectrode surfaces, as well as the migration of H^+^ through the membrane; the arrow labeled e^−^ indicates the electron flow in the external circuit; vertical double-headed arrows indicate the band gaps (*E*_g_); dashed lines mark the H^+^/H_2_ and O_2_/H_2_O redox levels; the sun symbol indicates illumination through the WO_3_ photoanode.

Based on these experimental values, the energy-level diagram of the tandem cell was constructed (Figure 3). For n-type WO_3_, the conduction band (CB) edge was placed slightly above *E*_fb_ (+0.30 V vs. RHE), as commonly assumed for moderately doped n-type oxides, and the valence band (VB) edge was obtained by adding *E*_g_ (+3.05 V vs. RHE). For p-type CuFe_2_O_4_, the VB edge was approximated by *E*_fb_ (+0.82 V vs. RHE), and the CB edge was obtained by subtracting *E*_g_ (−0.61 V vs. RHE). The VB of WO_3_ lies ≈1.8 V below the O_2_/H_2_O level, ensuring a large thermodynamic driving force for the OER, whereas the CB of CuFe_2_O_4_ lies ≈0.6 V above the H^+^/H_2_ level, so that photogenerated electrons can reduce protons. Majority carriers, i.e., electrons in WO_3_ and holes in CuFe_2_O_4_, recombine through the external circuit, while the protons released at the photoanode migrate through the Nafion 117 membrane towards the photocathode.

Most importantly, the flat-band potential of the photocathode (+0.82 V vs. RHE) is more positive than that of the photoanode (+0.38 V vs. RHE). This defines a potential window of ≈0.4 V in which both photoelectrodes can, in principle, generate photocurrent simultaneously, which is the necessary condition for the bias-free operation of a tandem cell [14]. A similar situation was reported for the WO_3_–CuCrO_2_ tandem, whose photocurrent onsets were located at 0.63 and 0.81 V vs. RHE for the photoanode and the photocathode, respectively [24]. By contrast, in our hematite/CuO device the CB of CuO lay slightly below the H^+^/H_2_ level, and an external bias was required to reach technologically relevant current densities [43]. In practice, surface recombination and kinetic overpotentials shift the photocurrent onsets with respect to *E*_fb_, reducing the effective overlap window and, consequently, the current at the operating point.

### 3.3. Photoelectrochemical Performance at the Beginning of Test

**Figure 4:**
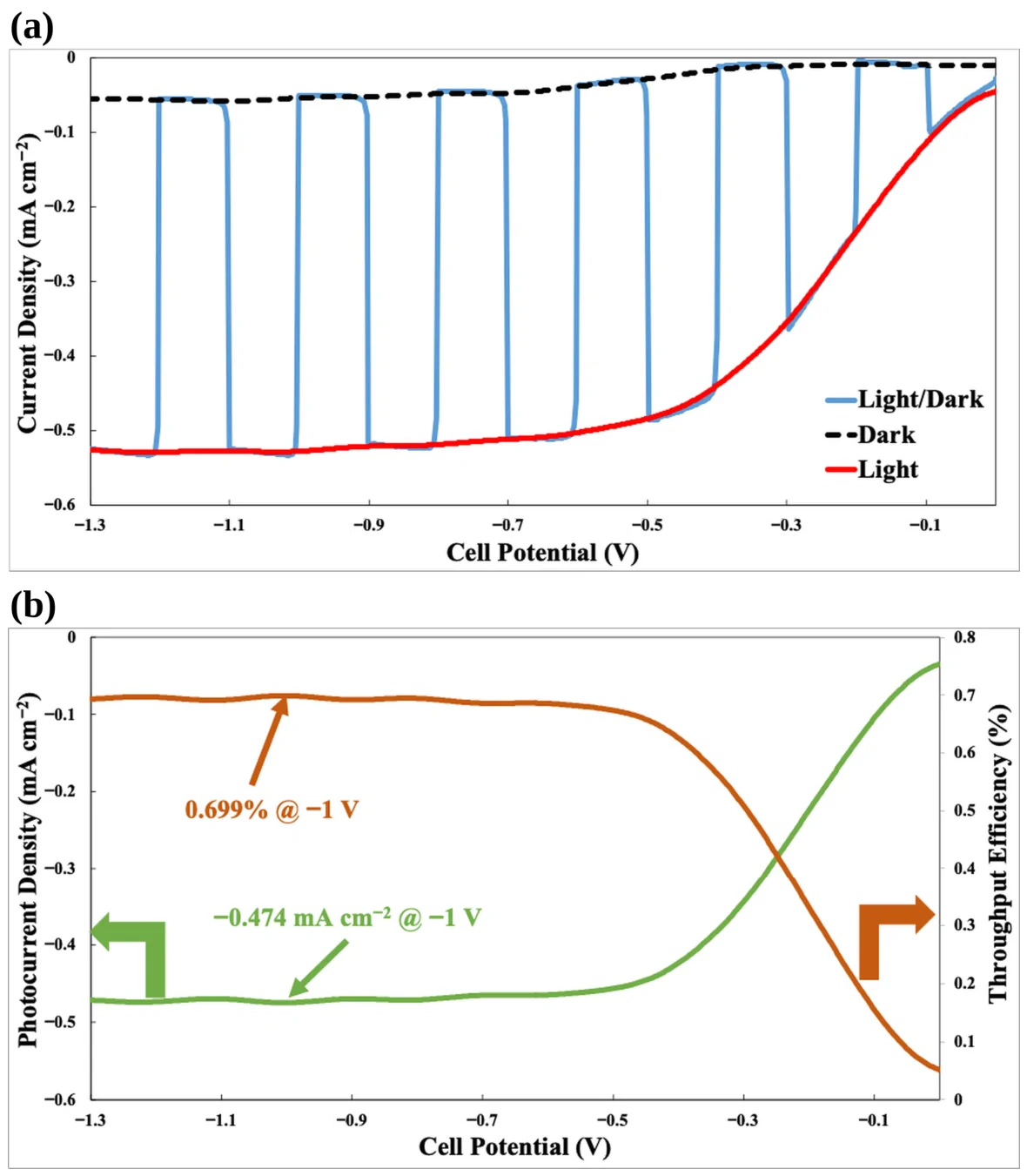
(**a**) Current density–voltage (*J*–*V*) curves of the fresh WO_3_/Nafion 117/CuFe_2_O_4_ tandem cell at the beginning of test (BoT), recorded in the dark, under continuous AM 1.5G illumination (100 mW cm^−2^) and under chopped illumination (5 s light/5 s dark) at a scan rate of 20 mV s^−1^. (**b**) Photocurrent density (*j*_p_ = *j*_light_ − *j*_dark_, left axis) and throughput efficiency (*η*_tp_, right axis; Equation (1)) as a function of the cell voltage, derived from the curves in (**a**).

Figure 4a shows the dark and illuminated *J*–*V* curves of the fresh tandem cell at BoT. In the dark, the current density remains below 0.06 mA cm^−2^ in absolute value over the whole voltage range (≈−0.01 mA cm^−2^ at 0 V and ≈−0.055 mA cm^−2^ at −1.3 V), indicating that no appreciable dark electrolysis takes place with these co-catalyst-free oxide electrodes, even at an assisting bias of 1.3 V. Under AM 1.5G illumination, the device exhibits a clear photocathodic response, with a current density of ≈−0.045 mA cm^−2^ already at 0 V, which demonstrates that the cell is able to operate without external bias, as anticipated by the energy-level diagram. Upon moving to more negative cell voltages, the current density increases steeply between −0.1 and −0.5 V and then levels off at ≈−0.51 to −0.53 mA cm^−2^ for cell voltages more negative than ≈−0.7 V. The chopped-light curve (5 s light/5 s dark) closely follows the dark and continuous-light curves, with sharp, nearly rectangular transients, indicating a fast photoresponse without pronounced transient spikes. It should be noted that, according to the photodiode convention adopted for the two-electrode measurements (Section 2.5), the assisting bias increases from right to left and the hydrogen-evolution current is negative; in this representation, the photocurrent onset at 0 V, the sigmoidal rise and the plateau are the expected features of a photon-limited tandem device, whose current is fixed by the absorbed photon flux once the applied bias compensates for the kinetic and recombination losses [14]. Consistently, the light and dark segments of the chopped-light curve coincide with the continuous-light and dark curves, respectively, which confirms the reproducibility of the measurements.

From a mechanistic standpoint, the saturation of the photocurrent indicates that, beyond ≈−0.6 V, the current is limited by the generation and separation of charge carriers in the photoelectrodes, i.e., by the absorbed photon flux and the internal quantum efficiency of the limiting absorber, rather than by the applied bias or by ohmic losses. This behavior differs markedly from that of our 10 cm^2^ hematite/CuO prototype with an anion-exchange membrane, in which the current density increased almost linearly with bias up to ≈−25 mA cm^−2^ at −1.3 V, i.e., in a predominantly bias-driven, ohmic-limited regime [43]. In the present WO_3_/CuFe_2_O_4_ device, the current is predominantly sustained by illumination, the dark current being only ≈10% of the total current even at −1.3 V, and the external bias mainly assists charge separation.

The photocurrent density (*j*_p_) and the throughput efficiency (*η*_tp_, Equation (1)) are reported in Figure 4b as a function of the cell voltage. At −1.0 V, *j*_p_ = −0.474 mA cm^−2^ and *η*_tp_ reaches 0.699%, with a plateau of ≈0.68–0.70% for cell voltages more negative than ≈−0.6 V, whereas *η*_tp_ decreases to ≈0.05% at 0 V. Because the electrical power supplied in the plateau region (|*j*_p_*V*_bias_| ≤ 0.62 mW cm^−2^) is lower than 1% of the incident optical power, *η*_tp_ is essentially determined by the saturated photocurrent. On this basis, −0.7 V, which corresponds to the onset of the photocurrent plateau, was selected as the bias-assisted operating point for the durability test, together with the bias-free condition (0 V).

### 3.4. Impedance Analysis at the Beginning of Test

**Figure 5:**
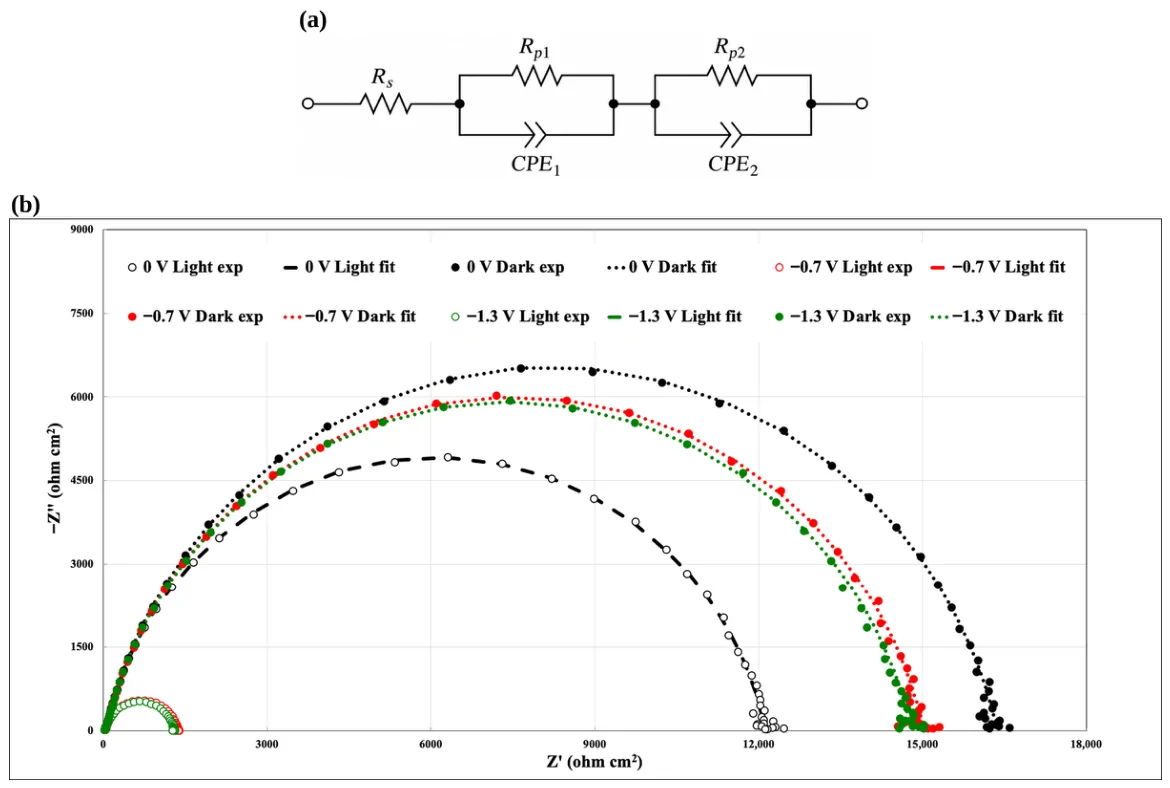
(**a**) Equivalent circuit, *R*_s_(*R*_p1_‖CPE_1_)(*R*_p2_‖CPE_2_), used to fit the impedance spectra. (**b**) Nyquist plots of the fresh tandem cell at the beginning of test (BoT), recorded in the dark and under AM 1.5G illumination (100 mW cm^−2^) at cell voltages of 0, −0.7 and −1.3 V. Symbols represent experimental data and lines represent the equivalent-circuit fits. The spectra recorded under illumination at −0.7 and −1.3 V, which have a much smaller impedance, appear as the small arcs at *Z*′ < 1500 Ω cm^2^; the corresponding fits (red and green dashed lines) largely overlap with the symbols, and the same spectra are shown on an expanded scale in Figure 7b.

Figure 5b reports the Nyquist plots of the fresh cell recorded at 0, −0.7 and −1.3 V, both in the dark and under AM 1.5G illumination. All spectra consist of a small high-frequency arc followed by a large, moderately depressed low-frequency semicircle. Upon illumination, the diameter of the low-frequency semicircle decreases markedly, particularly at −0.7 and −1.3 V, reflecting the contribution of photogenerated carriers to the interfacial reactions. The spectra were fitted with the *R*_s_(*R*_p1_‖CPE_1_)(*R*_p2_‖CPE_2_) equivalent circuit shown in Figure 5a, and the fitting curves closely reproduce the experimental data. The extracted parameters are listed in Table 1, and the characteristic time constants of the two *R*‖CPE elements, calculated with Equation (4), are reported in Table S1.

**Table 1:** Equivalent-circuit parameters obtained by fitting the EIS spectra of the fresh tandem cell at the beginning of test (BoT) with the *R*_s_(*R*_p1_‖CPE_1_)(*R*_p2_‖CPE_2_) circuit shown in Figure 5a.

Condition	*R*_s_ (Ω cm^2^)	*R*_p1_ (Ω cm^2^)	*Y*_1_ (µS cm^−2^ s*^n^*)	*n*_1_	*R*_p2_ (Ω cm^2^)	*Y*_2_ (µS cm^−2^ s*^n^*)	*n*_2_
0 V, dark	15.2	36.0	0.166	0.95	16,316.8	0.263	0.86
0 V, light	14.9	28.0	0.214	0.96	12,083.1	0.292	0.87
−0.7 V, dark	14.8	32.0	0.187	0.91	14,980.2	0.274	0.86
−0.7 V, light	15.1	20.0	0.300	0.92	1333.9	1.92	0.88
−1.3 V, dark	15.0	30.0	0.200	0.94	14,755.0	0.266	0.86
−1.3 V, light	14.7	18.0	0.333	0.85	1263.3	1.80	0.88

Conditions indicate the cell voltage and whether the spectra were recorded in the dark or under AM 1.5G illumination (100 mW cm^−2^). *Y* and *n* are the parameters of the constant phase elements, *Z*_CPE_ = 1/[*Y*(*jω*)*^n^*] (Equation (3)); 1 µS cm^−2^ s*^n^* = 10^−6^ S cm^−2^ s*^n^*.

The series resistance *R*_s_ (14.7–15.2 Ω cm^2^) remains constant regardless of illumination and cell voltage. This resistance is largely determined by the protonic resistance of the hydrated membrane and by the electronic resistance of the FTO current collectors and contacts, and its value is comparable to that measured for the 10 cm^2^ anion-exchange membrane prototype (≈16 Ω cm^2^) [43]. However, at the low current densities of the present device, the associated ohmic drop is only ≈7–8 mV at the photocurrent plateau, confirming that ohmic losses are negligible in this regime, in agreement with the photo-limited shape of the *J*–*V* curves.

The high-frequency element (*R*_p1_‖CPE_1_) shows small resistances (18–36 Ω cm^2^) and time constants in the microsecond range (*τ*_1_ ≈ 0.7–3.6 µs, Table S1), with *R*_p1_ decreasing from 36.0 Ω cm^2^ (dark, 0 V) to 18.0 Ω cm^2^ (light, −1.3 V). This contribution is attributed to fast charge-transport processes within the oxide films and across the solid–solid contacts, namely the oxide/FTO and oxide/membrane interfaces. The low-frequency element (*R*_p2_‖CPE_2_) accounts for more than 97% of the total resistance and governs the device response. In the dark, *R*_p2_ is very large (14.8–16.3 kΩ cm^2^) and almost independent of the cell voltage, consistent with the negligible dark currents. Under illumination, *R*_p2_ decreases by ≈26% at 0 V (12.1 kΩ cm^2^) and by ≈91% at −0.7 and −1.3 V (1.33 and 1.26 kΩ cm^2^, respectively), while the CPE_2_ parameter *Y*_2_ increases approximately 7-fold, from ≈0.27 to 1.92 and 1.80 µS cm^−2^ s*^n^*. The simultaneous decrease in *R*_p2_ and increase in the associated capacitance under illumination is the typical signature of interfacial charge transfer mediated by photogenerated carriers accumulated in surface states, as described for hematite [67,68] and CaFe_2_O_4_ [36] photoelectrodes. Therefore, the low-frequency element is assigned to the interfacial charge-transfer processes, i.e., water oxidation at WO_3_ and proton reduction at CuFe_2_O_4_, with characteristic time constants of ≈1.6–1.8 ms in the dark and ≈1.0–1.1 ms under illumination at −0.7 and −1.3 V (Table S1). At 0 V under illumination, the limited decrease in *R*_p2_ reflects the small driving force available at the bias-free operating point, where recombination competes effectively with interfacial charge transfer.

It should be noted that, because the two photoelectrodes are connected in series, the anodic and cathodic interfacial impedances add up, and each *R*‖CPE element may lump contributions from both electrodes. Overall, the impedance analysis complements the *J*–*V* data by quantifying the relative weight of the ohmic and kinetic contributions, and it identifies interfacial charge transfer, rather than series resistance, as the main loss mechanism of the device.

### 3.5. Durability Under Bias-Free and Bias-Assisted Operation

**Figure 6:**
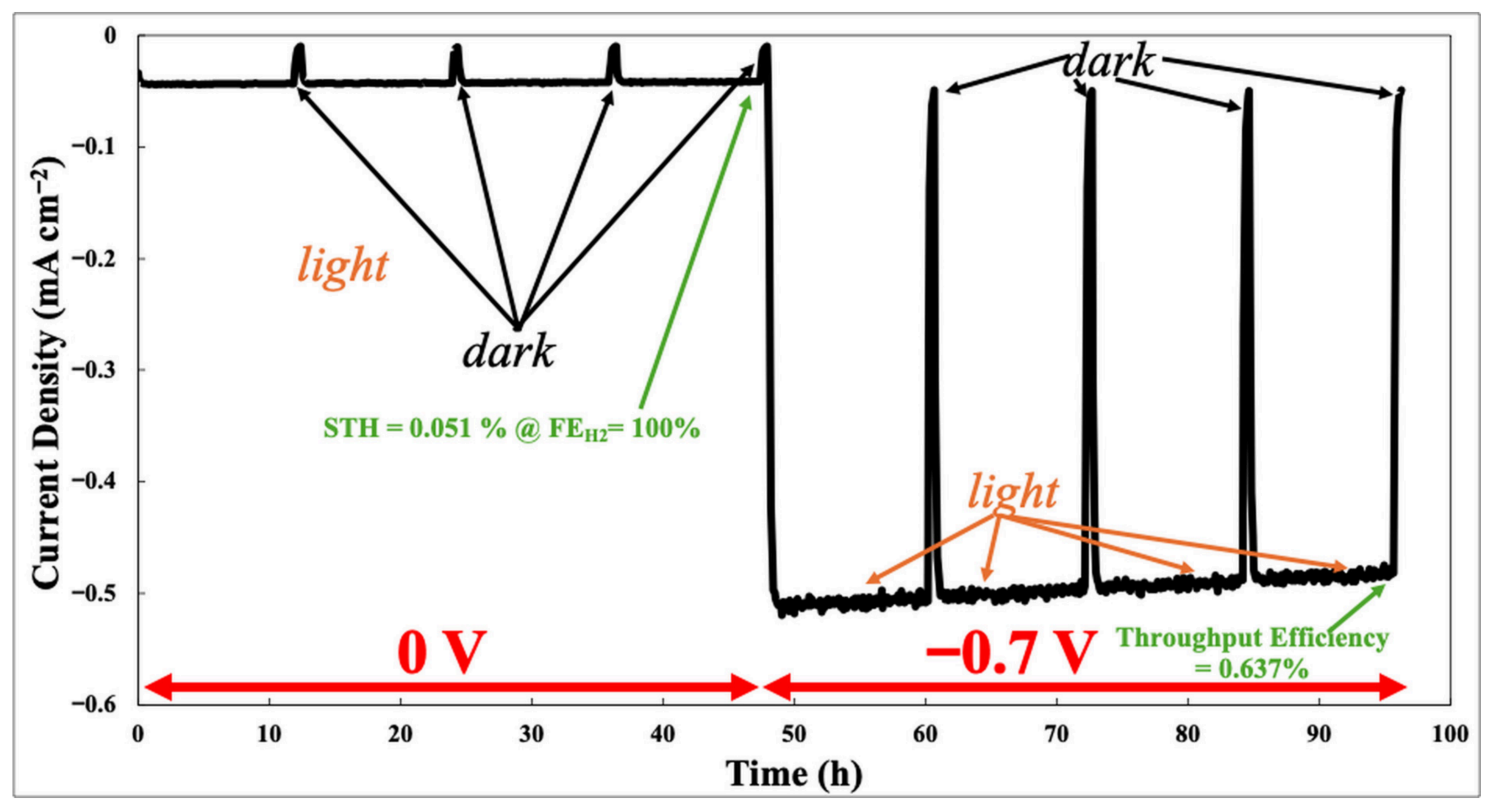
Potentiostatic durability test of the tandem cell under AM 1.5G illumination (100 mW cm^−2^): 48 h at 0 V (bias-free operation) followed by 48 h at −0.7 V. Thirty-minute dark intervals were introduced every 12 h as internal reference conditions. The solar-to-hydrogen efficiency at 0 V (STH, Equation (2), calculated assuming a Faradaic efficiency for H_2_ evolution of 100%) and the throughput efficiency at the end of the −0.7 V stage (*η*_tp_, Equation (1)) are indicated.

The long-term stability of the tandem cell was evaluated by chronoamperometry under continuous AM 1.5G illumination for 96 h, according to the two-stage protocol shown in Figure 6: 48 h at 0 V (bias-free operation) followed by 48 h at −0.7 V, i.e., at the onset of the photocurrent plateau (Figure 4a), where the device delivers almost its saturated photocurrent with a minimum assisting bias. Thirty-minute dark intervals were introduced every 12 h as internal reference conditions.

At 0 V, the current density decreases only slightly, from ≈−0.044 to ≈−0.042 mA cm^−2^ over 48 h. The dark intervals bring the current to ≈−0.011 mA cm^−2^, and the initial value is immediately recovered upon re-illumination, indicating that the light/dark cycles do not cause irreversible losses. Since the cell was continuously surrounded by flowing N_2_, the reduction of atmospheric O_2_ can be excluded; the small cathodic current recorded in the dark at 0 V, which cannot originate from water splitting, is ascribed to the discharge of the charge accumulated in WO_3_ under illumination (W^5+^/H_x_WO_3_ centers, Section 3.9) and/or to the reduction of traces of photogenerated O_2_ retained in the hydrated membrane. Its contribution is, however, marginal on the time scale of the test: the charge passed during each 30 min dark interval (≈0.02 C cm^−2^) is negligible compared with the ≈7.3 C cm^−2^ passed at 0 V over 48 h and with the ≈93 C cm^−2^ passed over the whole protocol.

Because the photocurrent of oxide photocathodes may also originate from their photoinduced self-reduction [33,34,39], a charge balance was performed to assess the possible contribution of photocorrosion to the measured current. The CuFe_2_O_4_ film contains at most ≈9.5 µmol of Cu and ≈19 µmol of Fe, i.e., the amounts contained in the 1 mL of precursor solution sprayed (Section 2.3). The complete Cu^2+^→Cu^+^ reduction in the film would therefore require ≈0.92 C cm^−2^, and even the complete reduction of all Cu^2+^ and Fe^3+^ cations to the metallic state, which is not observed (Section 3.8 and Section 3.9), would require at most ≈7.4 C cm^−2^, i.e., less than 8% of the ≈93 C cm^−2^ passed during the whole protocol. At −0.7 V, the Cu^2+^ → Cu^+^ reduction capacity of the film would be exhausted in about 30 min, and its complete reduction to the metallic state in about 4 h, whereas the current was sustained at ≈0.5 mA cm^−2^ for 48 h with a decrease of only ≈6%. In addition, since the two photoelectrodes are connected in series, the same charge must flow through the WO_3_ photoanode, in which tungsten is already in its highest oxidation state (W^6+^); the anodic charge therefore cannot be sustained by the self-oxidation of the photoanode and must be associated with the oxidation of water to O_2_ and, possibly, to peroxo species [32]. Hence, photocorrosion can account for only a minor fraction of the charge passed, and the current must be sustained predominantly by faradaic reactions involving water. Nevertheless, photocorrosion does occur to a limited extent, as shown by the partial Cu^2+^ → Cu^+^ reduction detected by XPS (Section 3.9), and it contributes to the slow degradation of the photocathode.

The corresponding STH efficiency (Equation (2)), calculated assuming a Faradaic efficiency of 100% [54], is 0.051%; if only the light-induced component of the current (*j*_light_ − *j*_dark_ ≈ 31 µA cm^−2^) is considered, a conservative STH value of ≈0.04% is obtained. Although modest in absolute terms, this bias-free current density is about four times higher than that reported for the WO_3_–CuCrO_2_ tandem operated in a two-compartment cell with liquid 0.1 M HClO_4_ (≈10 µA cm^−2^), and it was sustained for a 16-fold longer time (48 h vs. 3 h) without any liquid electrolyte [24]. It should be noted that the H_2_ production was not quantified by gas chromatography in this study; therefore, the STH values must be regarded as upper estimates. The crossover of photogenerated O_2_ through the hydrated membrane and the formation of peroxide species at WO_3_ in acidic media, which lowered the O_2_ Faradaic efficiency of a WO_3_/Nafion photoelectrochemical cell to 47–80% [50,51], may indeed reduce the Faradaic efficiency. In the present 1 cm^2^ configuration without housing, the gases evolved at the two electrodes are released at the edges of the photoelectrolysis cell into the surrounding N_2_ atmosphere and can be neither separated nor collected quantitatively; moreover, the expected production rates are very small (≈0.8 µmol h^−1^ of H_2_ at 0 V and ≈9 µmol h^−1^ at −0.7 V, assuming unit Faradaic efficiency), and the H_2_ produced under bias-free conditions, continuously diluted in the surrounding N_2_ flow, falls below the detection limit of the available gas-chromatographic set-up. The direct quantification of the Faradaic efficiency is therefore deferred to the scaled-up, housed version of the device (active area of at least 10 cm^2^), as previously done for our alkaline prototype, for which a H_2_ purity of 99.95% was measured at the cathode outlet [43].

At −0.7 V, the current density decreases gradually from ≈−0.514 to ≈−0.482 mA cm^−2^ over 48 h (≈6%, i.e., ≈0.13% h^−1^), while the dark current remains at ≈−0.05 mA cm^−2^. At the end of the test, *j*_p_ ≈ −0.43 mA cm^−2^ and *η*_tp_ = 0.637%. The trace shows no sudden drops, spikes or irreversible losses, which excludes catastrophic failure modes such as membrane dehydration, loss of electrical contact or gas-induced separation of the cell components. Importantly, the smooth decay points to slow, spatially distributed degradation processes rather than to localized failures. From an engineering perspective, this is an encouraging indication, since slow materials-related degradation can be tackled by electrode and interface optimization without changing the cell concept. Such stability is noteworthy, considering that the cell was operated without housing, with water supplied only by periodic wetting of the membrane edges, and is consistent with the unchanged series resistance at EoT (Section 3.6).

### 3.6. End-of-Test Performance and Impedance Evolution

**Figure 7:**
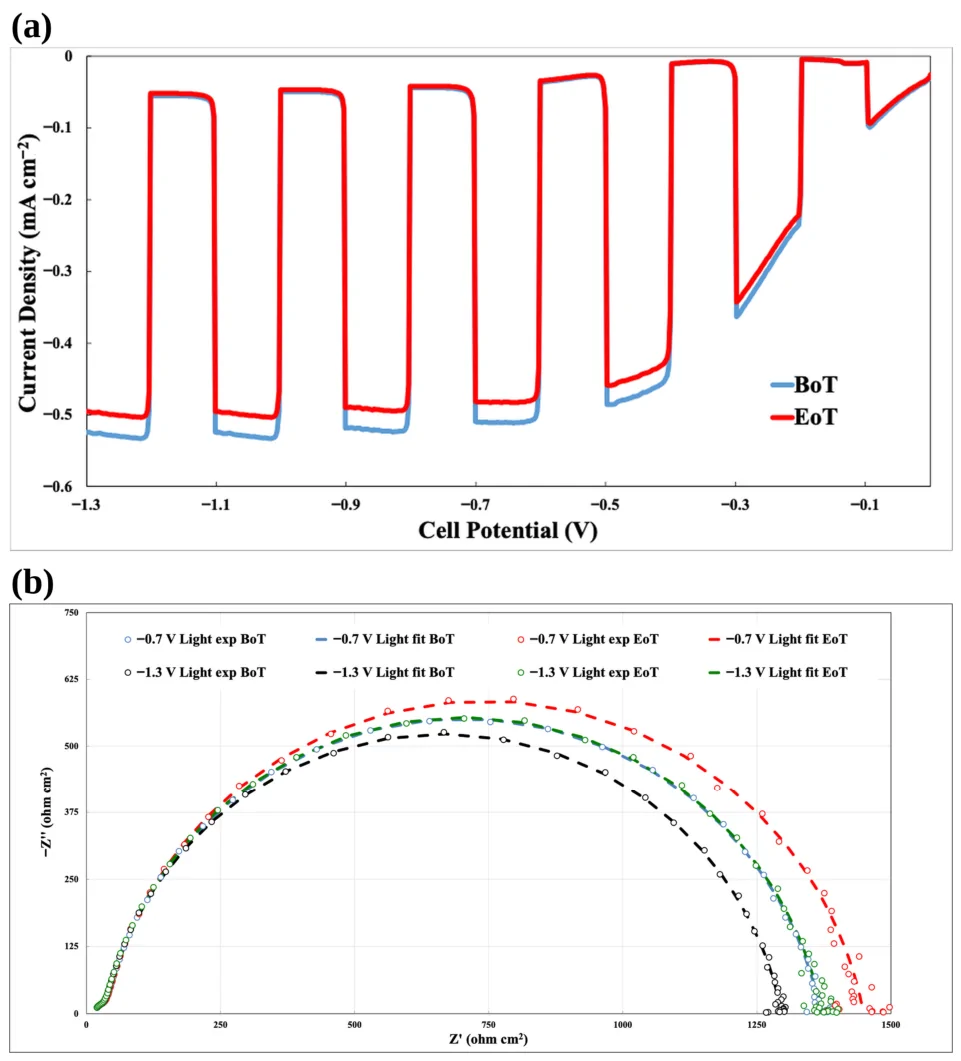
Comparison between the beginning of test (BoT) and the end of test (EoT, after the 96 h durability protocol): (**a**) chopped-light *J*–*V* curves under AM 1.5G illumination (100 mW cm^−2^; scan rate 20 mV s^−1^); (**b**) Nyquist plots recorded under illumination at −0.7 and −1.3 V. In (**b**), symbols represent experimental data and dashed lines represent the equivalent-circuit fits.

After the 96 h protocol, the cell was characterized again under the same conditions used at BoT (Figure 7 and Figure S2). The saturated current density under illumination decreased from ≈−0.53 to ≈−0.50 mA cm^−2^ at −1.3 V (Figure 7a); at −1.0 V, *j*_p_ decreased from −0.474 to −0.448 mA cm^−2^ (−5.5%) and *η*_tp_ from 0.699% to 0.66% (−5.6%). Conversely, the BoT and EoT curves are almost superimposed in the low-bias region (0 to −0.3 V), and the dark current is unaffected. The degradation therefore mainly affected the saturated photocurrent rather than the photocurrent onset, which is consistent with a partial loss of photoactive area or of light-harvesting capability of the limiting photoelectrode, rather than with a shift in the band-edge energetics.

The impedance spectra at EoT (Figure 7b and Figure S3) were fitted with the same equivalent circuit, and the resulting parameters are listed in Table 2. *R*_s_ remains unchanged (14.9–15.3 Ω cm^2^), confirming that the hydration of the membrane and the quality of the contacts were preserved throughout the test, while *R*_p1_ increases only slightly (+3–5%). Under illumination, *R*_p2_ increases by ≈6% at both −0.7 V (from 1333.9 to 1418.9 Ω cm^2^) and −1.3 V (from 1263.3 to 1343.1 Ω cm^2^), in close agreement with the ≈5.5% decrease in the saturated photocurrent. In the dark, on the other hand, *R*_p2_ decreases by ≈3% at all cell voltages. The CPE parameters *Y*_1_ and *Y*_2_ increase in all conditions (+8–10% and +18–26%, respectively), indicating a larger electrochemically active interface and/or a higher density of surface states, in agreement with the surface hydroxylation and the formation of reduced W^5+^ and Cu^+^ species revealed by XPS (Section 3.9). The light-induced decrease in *R*_p2_ at −0.7 and −1.3 V remains ≈90% at EoT (vs. ≈91% at BoT), showing that the photoactive charge-transfer mechanism is preserved, whereas the slight decrease in the dark *R*_p2_ suggests the formation of additional parallel pathways, such as recombination through surface states, which do not contribute to the photocurrent. The main performance and impedance descriptors at BoT and EoT are summarized in Table 3.

### 3.7. Distribution of Relaxation Times

**Figure 8:**
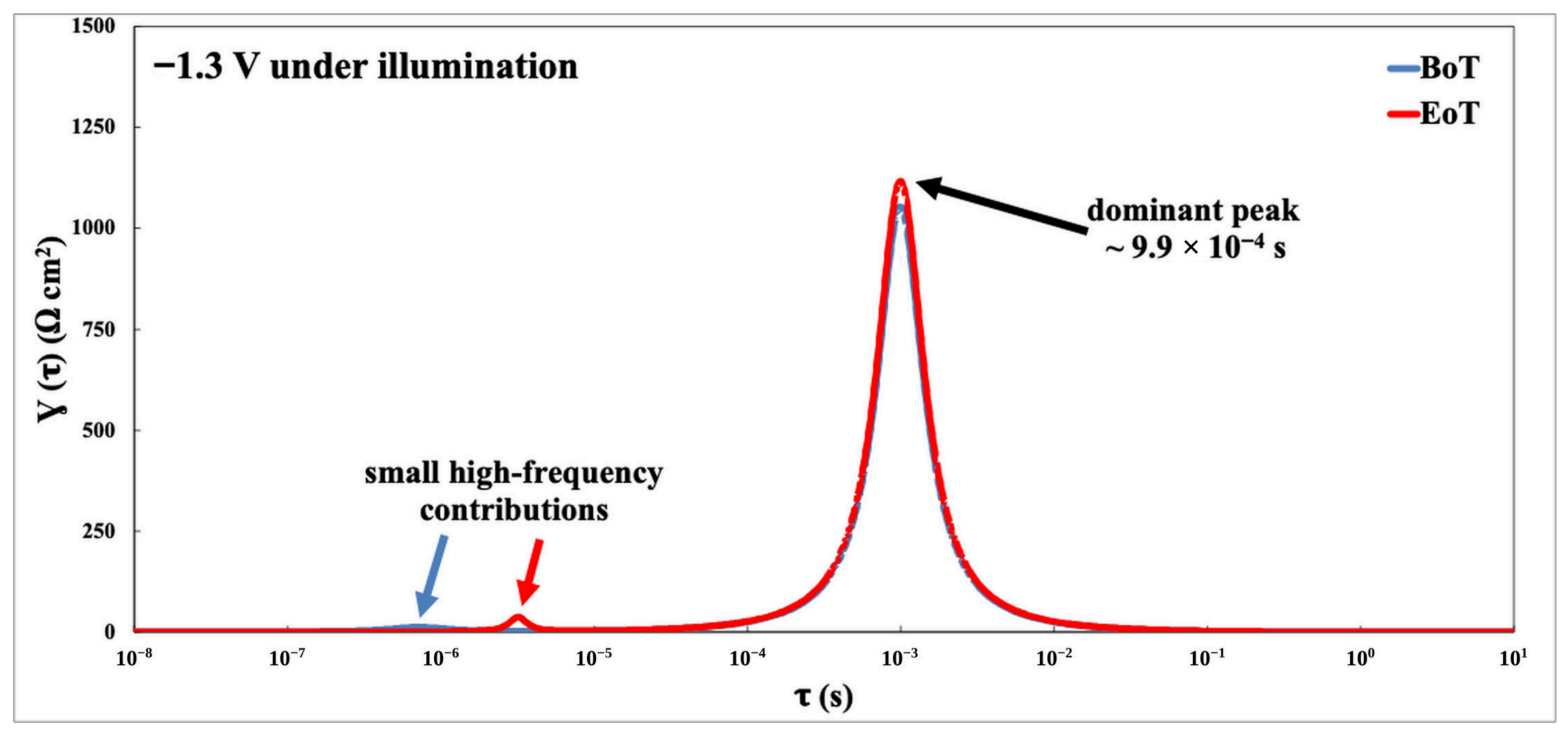
Distribution of relaxation times (DRT) profiles extracted from the EIS spectra recorded under AM 1.5G illumination at −1.3 V at the beginning of test (BoT) and at the end of test (EoT, after 96 h). The dominant relaxation process is centered at ≈9.9 × 10^−4^ s, with minor high-frequency contributions in the microsecond range.

Figure 8 compares the DRT profiles extracted from the spectra recorded under illumination at −1.3 V, i.e., at the operating condition with the highest photocurrent. At both BoT and EoT, the distribution is dominated by a single, well-defined peak in the millisecond domain, centered at *τ* ≈ 9.9 × 10^−4^ s and attributed to interfacial charge-transfer and double-layer dynamics. After the durability test, the amplitude of this peak increases from ≈1.06 × 10^3^ to ≈1.12 × 10^3^ Ω cm^2^ (+6%), whereas its position remains essentially unchanged. Minor high-frequency contributions, with amplitudes below 50 Ω cm^2^, appear at ≈0.8 µs at BoT and at ≈3 µs at EoT.

These features are fully consistent with the equivalent-circuit analysis: the time constants calculated from the fitted parameters with Equation (4) are *τ*_2_ = 0.99 and 1.28 ms and *τ*_1_ = 0.72 and 3.2 µs at BoT and EoT, respectively (Table S1). The small difference between the *τ*_2_ values obtained by the two methods is within the uncertainty arising from the correlation of the CPE parameters and from the smoothing introduced by the regularization [56]. Collectively, these results show that the device response is governed by a millisecond-scale interfacial charge-transfer process, while the microsecond process is associated with the high-frequency *R*_p1_‖CPE_1_ element. The shift in the latter towards longer relaxation times at EoT is consistent with the increase in *Y*_1_ and *n*_1_ (Table 1 and Table 2) and may reflect the modification of the solid–solid contacts, namely the ionomer residue on WO_3_ and the local detachment of CuFe_2_O_4_ discussed in Section 3.9 and Section 3.10. Notably, the dominant relaxation of the present device is about one order of magnitude faster than that observed for the hematite/CuO prototype (*τ* ≈ 10^−2^ s) [43].

It is worth noting that the smoothing imposed by the Tikhonov regularization can merge closely spaced relaxations; hence, as for the equivalent-circuit analysis, the main peak may comprise partially overlapping contributions of the photoanode and photocathode interfaces [48]. Nevertheless, the agreement between the model-free DRT and the model-based equivalent-circuit approach supports the robustness of the assignment.

### 3.8. Structural Analysis by X-Ray Diffraction

The XRD patterns of the as-prepared (fresh) and used photoelectrodes are shown in Figure S4. The fresh WO_3_ film shows the expected reflections of monoclinic WO_3_ (PDF 43-1035), namely the (002), (020) and (200) triplet at ≈23.1°, 23.6° and 24.4°, the (112) reflection at ≈28.3° and the (140) reflection at ≈50.0°, while the (120) and (202)/(122) reflections overlap with the (110) and (101) reflections of the tetragonal SnO_2_ of the FTO substrate (PDF 72-1147). The fresh CuFe_2_O_4_ film exhibits weak and broad reflections at ≈31.0° and ≈36.4°, indexed to the (200) and (211) planes of tetragonal spinel CuFe_2_O_4_ (PDF 34-0425), together with the SnO_2_ reflections of the substrate. The low intensity of the spinel reflections is consistent with the small thickness and the nanocrystalline nature of the film (Section 3.10), and no reflections of CuO, Cu_2_O or iron oxides are detected.

After the durability test (Figure S4a), the WO_3_ reflections are strongly attenuated and broadened: the (002)/(020)/(200) triplet merges into a broad feature between ≈23.5° and 25°, and the (112) and (140) reflections are barely detectable, whereas the SnO_2_ reflections are retained. All reflections, including those of the substrate, are shifted by ≈+0.3–0.4°, which is attributed to a sample-height displacement rather than to a change in the lattice parameters. These changes indicate a loss of long-range crystalline order in the WO_3_ film, most plausibly induced by proton insertion and hydration in contact with the strongly acidic ionomer, which can reduce the lattice distortion and generate nanocrystalline disorder [25,29], in agreement with the increase in W^5+^ species detected by XPS (Section 3.9). Since the W signals in EDS are essentially retained (Figure S5) and the film coverage is preserved (Section 3.10), the attenuation of the reflections cannot be ascribed to a substantial loss of material.

For the used CuFe_2_O_4_ (Figure S4b), the (200) reflection is no longer visible, while the (211) reflection is still detectable, and no new reflections of metallic Cu, Cu_2_O, CuO or iron oxides appear. Hence, no bulk phase transformation of the photocathode occurred within the detection limit of XRD, and the weakening of the spinel reflections is consistent with the partial detachment of the film observed by SEM and/or with a near-surface loss of crystallinity. In short, the XRD comparison reveals a loss of long-range order in WO_3_ but no bulk phase transformation in either photoelectrode, in sharp contrast with the behavior of CuO in our anion-exchange membrane device, whose reflections almost disappeared after 240 h because of electro-reduction to Cu_2_O/Cu [43].

### 3.9. Surface Chemistry by X-Ray Photoelectron Spectroscopy

To complement the bulk-sensitive XRD analysis, surface-sensitive XPS measurements were performed on both photoelectrodes. The high-resolution spectra of the WO_3_ photoanode before and after the durability test are shown in Figure S6. The surface of the fresh electrode is dominated by W^6+^, with W 4f_7/2_ and W 4f_5/2_ components at 35.6 and 37.7 eV; a minor W^5+^ doublet at lower binding energy (≈34.5 and ≈36.7 eV) indicates a small concentration of reduced tungsten and oxygen-deficient surface sites, and the W 5p_3/2_ contribution appears at ≈41.6 eV. After 96 h of operation, W^6+^ remains dominant, while the W^5+^ contribution increases moderately. The O 1s region is resolved into lattice oxygen (≈530.2 eV), hydroxyl/oxygen-vacancy-related oxygen (OH/O_v_, ≈531.5 eV) and higher-binding-energy adsorbed species (≈532.8 eV); after testing, the lattice-oxygen fraction decreases and both the OH/O_v_ and the adsorbed-species contributions increase. Overall, these changes are compatible with a mild surface reduction and hydration rather than with a wholesale transformation of the WO_3_ phase.

Because the used WO_3_ electrode was in direct contact with Nafion, the high-binding-energy O 1s contribution cannot be assigned exclusively to adsorbed water or intrinsic WO_3_ defects. The C 1s, F 1s and S 2p regions (Figure S7) reveal, on the used WO_3_ surface, a CF_2_-related C 1s component at 292.1 eV, an F 1s peak at ≈688.8 eV (C–F_x_) and an S 2p doublet with the 2p_3/2_ component at ≈168.6 eV (SO_3_^−^), which provide an internally consistent signature of residual ionomer [65]; accordingly, the F Kα line is also detected by EDS on the used WO_3_ (Figure S5). The sulfonate and ether oxygens of the ionomer therefore contribute to the high-binding-energy O 1s intensity of the used electrode. Since Nafion is prone to X-ray-induced degradation during XPS acquisition [65], the ionomer-related signals were evaluated only qualitatively. Notably, the presence of an ionomer layer firmly adhered to WO_3_ testifies to the intimate contact established at the photoanode/membrane interface in the zero-gap configuration.

**Figure 9:**
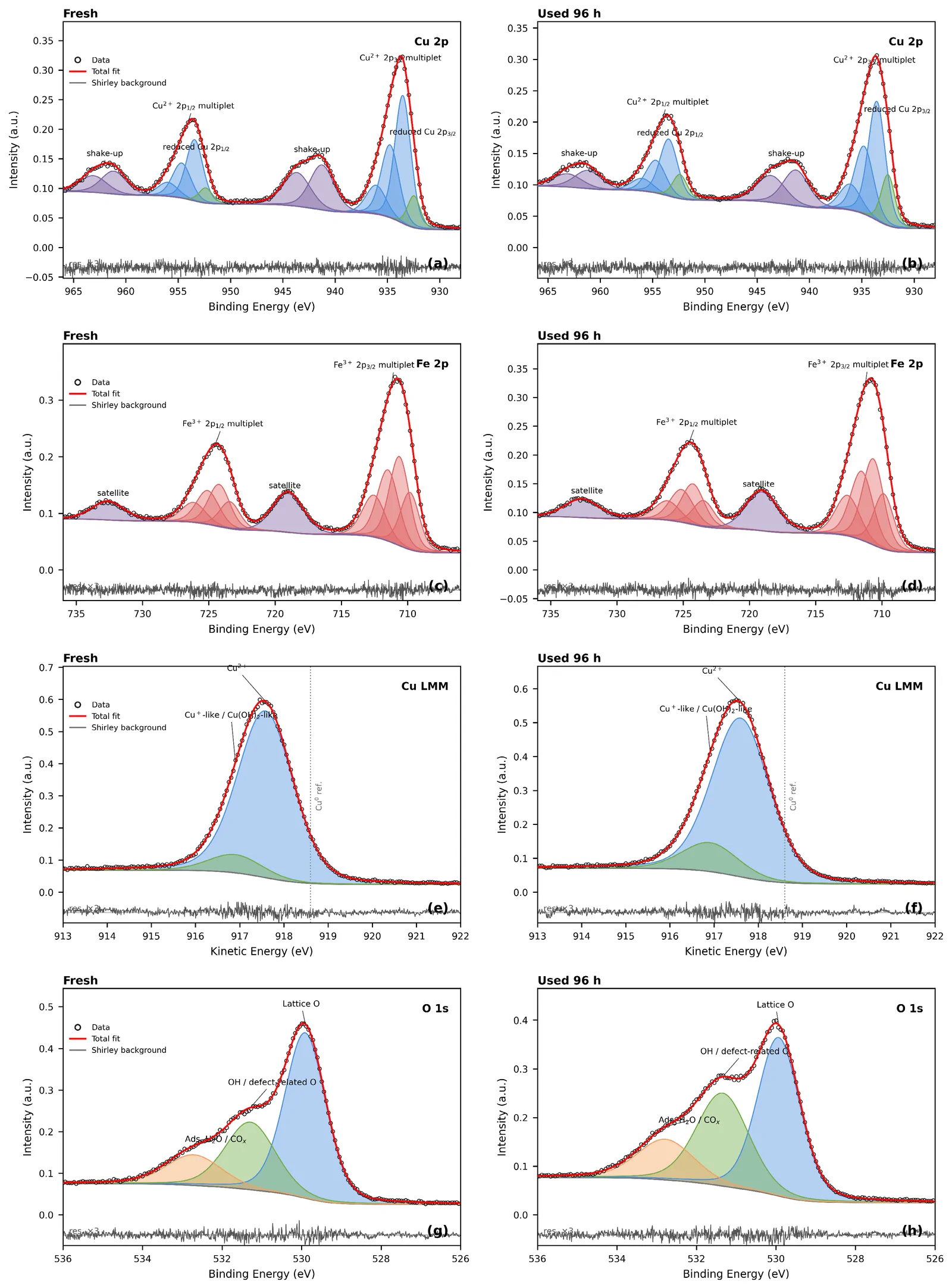
High-resolution XPS spectra and peak deconvolution of the CuFe_2_O_4_/FTO photocathode before electrochemical testing (fresh, left) and after the 96 h durability test (used, right): (**a**,**b**) Cu 2p region, fitted with the broad Cu^2+^ multiplet envelope and shake-up satellites together with a lower-binding-energy reduced-Cu contribution; (**c**,**d**) Fe 2p region, described by an Fe^3+^ multiplet model including the characteristic satellites near 719 and 733 eV, without assigning the low-binding-energy multiplet intensity to a separate Fe^2+^ species; (**e**,**f**) Cu LMM Auger region, used to distinguish Cu^+^-like from Cu^0^-like reduced copper; (**g**,**h**) O 1s region. Open circles: experimental data; red line: total fit; grey line: Shirley background; shaded areas: fitted components (blue: Cu^2+^ multiplet in (**a**,**b**,**e**,**f**) and lattice O in (**g**,**h**); green: reduced Cu in (**a**,**b**), Cu^+^-like/Cu(OH)_2_-like component in (**e**,**f**) and OH/defect-related O in (**g**,**h**); red: Fe^3+^ multiplet in (**c**,**d**); purple: shake-up and Fe satellites; orange: adsorbed H_2_O/CO_x_ in (**g**,**h**)); the residuals (×3) are shown at the bottom of each panel.

The XPS spectra of the CuFe_2_O_4_ photocathode (Figure 9) provide direct evidence of the evolution of its surface chemical state after operation. The Cu 2p region is described by the intrinsically broad Cu^2+^ multiplet envelope (Cu 2p_3/2_ at ≈933.7 eV) and its intense shake-up satellites at ≈940–945 eV, rather than by unrealistically narrow single components [62,69]. A lower-binding-energy reduced-Cu contribution (≈932.6 eV) is present; however, Cu 2p alone cannot discriminate Cu^+^ from Cu^0^. The Cu LMM Auger region was therefore analyzed: the reduced-Cu signal appears at a kinetic energy of ≈917.0 eV and is modeled as Cu^+^-like, whereas no separate Cu^0^ component (expected at ≈918.6 eV) is required [62]. The increase in the reduced-copper fraction from the fresh to the used electrode is accompanied by a decrease in the satellite-to-principal-line ratio, as expected when part of the Cu^2+^ population is reduced. The Fe 2p region is described by an Fe^3+^ multiplet envelope (Fe 2p_3/2_ at ≈711 eV) with the characteristic satellites at ≈719 and ≈733 eV; the low-binding-energy component at ≈709.8–710 eV lies within the normal Fe^3+^ multiplet structure and is therefore not assigned to Fe^2+^ [63,64]. The fresh and used Fe 2p envelopes remain very similar, supporting the retention of a predominantly Fe^3+^ surface chemistry during operation. The O 1s spectra corroborate this picture, showing a decrease in lattice oxygen (≈529.9 eV) and an increase in hydroxyl/defect-related oxygen (≈531.3 eV) after testing. In all cases, the fit residuals are comparable to the noise level.

Taken together, the modest increase in the Cu^+^-like component, the preserved Fe^3+^ multiplet, the unchanged Cu/Fe ratio indicated by EDS (Section 3.10) and the absence of new crystalline copper phases in XRD point to a limited near-surface reduction and hydroxylation of the photocathode rather than to its bulk decomposition. The partial reduction of Cu^2+^ is ascribed to photogenerated electrons that, instead of being transferred to protons, reduce lattice copper, a self-reduction pathway well known for copper-based photocathodes [33,34].

### 3.10. Morphology and Composition by SEM-EDS and TEM

**Figure 10:**
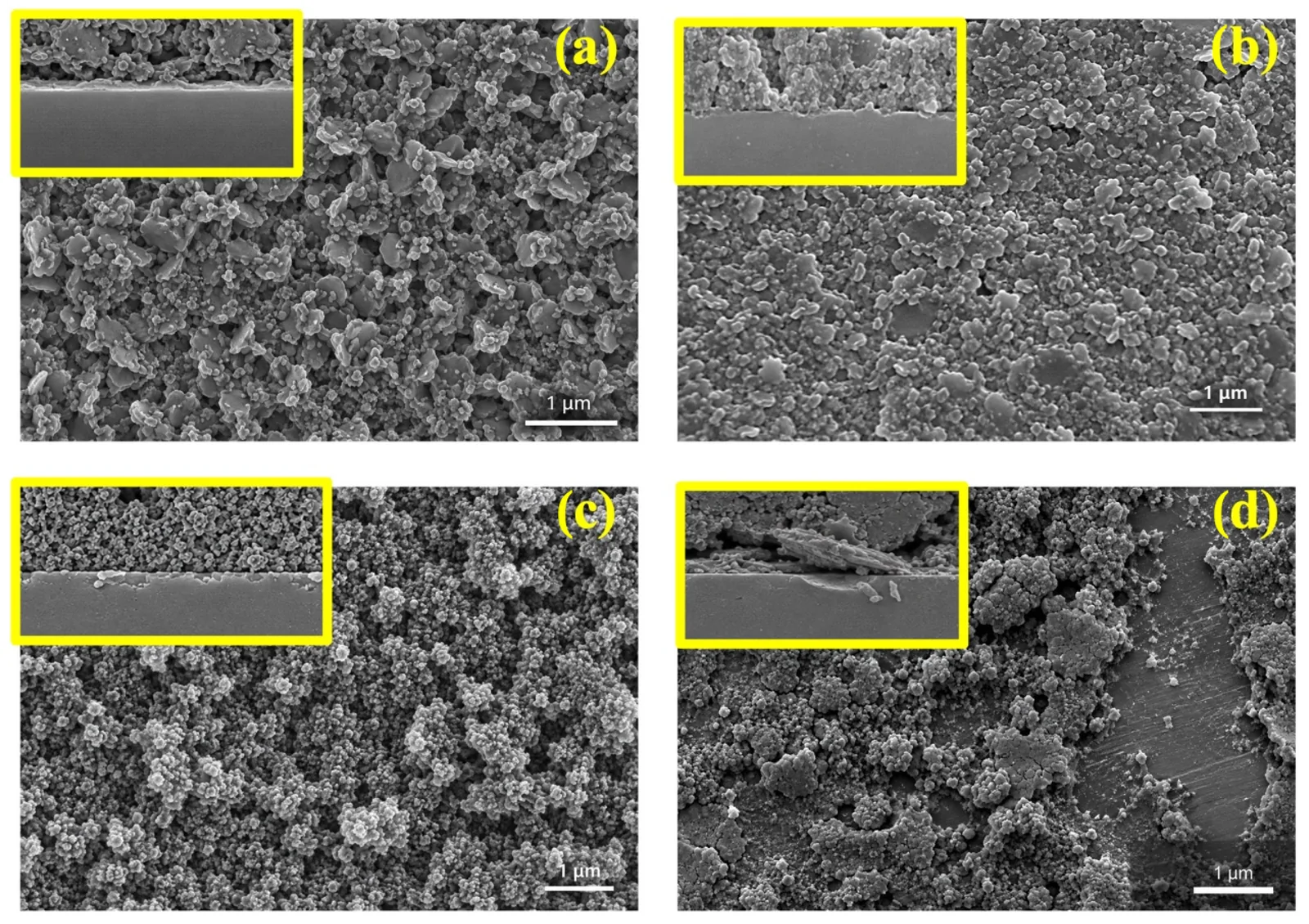
Field-emission scanning electron microscopy (FE-SEM) images of the photoelectrodes before electrochemical testing (fresh) and after the 96 h durability test (used): (**a**) fresh WO_3_/FTO; (**b**) used WO_3_/FTO; (**c**) fresh CuFe_2_O_4_/FTO; (**d**) used CuFe_2_O_4_/FTO. Insets show representative cross-sectional views of the corresponding films on the FTO glass.

FE-SEM images of the fresh and used photoelectrodes are shown in Figure 10. In the fresh WO_3_ film (Figure 10a), the surface is characterized by a porous network of nanoparticles of a few tens of nanometers, interspersed with platelet-like grains of a few hundred nanometers, which uniformly cover the FTO substrate; the cross-sectional view (inset) shows a continuous layer well adhered to the substrate. After 96 h of operation (Figure 10b), the WO_3_ layer preserves a continuous coverage of the substrate, highlighting its robust adhesion; however, subtle modifications are evident. The film appears more compact, with coalesced grains, smoother surfaces and fewer platelet-like features, consistent with the surface hydration and the loss of long-range order discussed above.

In contrast, the fresh CuFe_2_O_4_ film (Figure 10c) displays a highly porous morphology formed by agglomerated nanoparticles, which assemble into cauliflower-like clusters of a few hundred nanometers. This open structure is advantageous for the contact with the ionomer and for light harvesting. After extended testing, however, the used CuFe_2_O_4_ (Figure 10d) reveals extended regions in which the film has detached and the smooth FTO substrate is exposed, together with compacted agglomerates; the cross-sectional view (inset) shows lifted flakes of the active layer. Because the photocathode film was in intimate contact with the membrane, it cannot be excluded that part of the detachment occurred during the disassembly of the cell; nevertheless, the weak adhesion of CuFe_2_O_4_ to FTO is in itself a durability issue for zero-gap operation.

The EDS analysis (Figure S5) provides complementary information on the chemical composition of the films. The fresh WO_3_ shows the O Kα, W M and W L lines together with the Sn L lines of the substrate; after the test, an additional F Kα signal appears, confirming the presence of the ionomer residue detected by XPS, whereas the W signals are essentially retained. For CuFe_2_O_4_, the O, Fe and Cu lines are observed in both fresh and used electrodes, with an almost identical Cu Kα/Fe Kα intensity ratio (≈0.72), indicating that the Cu/Fe stoichiometry within the probed volume was not altered by operation.

TEM images (Figure S8) show that the fresh WO_3_ consists of nanocrystals of ≈10–20 nm, partly elongated or platelet-like, with well-defined lattice fringes (Figure S8a). After the test (Figure S8b), the WO_3_ particles appear more rounded and agglomerated (≈8–15 nm) and their edges are less defined, while lattice fringes are still visible in many particles, indicating that crystalline cores are retained beneath a more disordered surface, in agreement with the XRD broadening. The fresh CuFe_2_O_4_ consists of rounded nanoparticles of ≈5–15 nm forming aggregates, with visible lattice fringes (Figure S8c), whereas the used material shows denser aggregates with less distinct particle boundaries (Figure S8d). Overall, the combined SEM, EDS and TEM analysis demonstrates that WO_3_ preserves its coverage, composition and crystalline cores, with only surface modifications, whereas CuFe_2_O_4_ retains its stoichiometry but suffers from a local loss of adhesion to the substrate.

Because the photocathode operated in contact with a strongly acidic ionomer, its possible chemical corrosion, i.e., the leaching of Cu and Fe, deserves specific attention. Within the probed volume, EDS indicates an unchanged Cu/Fe intensity ratio, XPS shows a preserved Fe^3+^ multiplet, and XRD reveals no new crystalline phases; together with the charge balance discussed in Section 3.5, these results indicate that the preferential leaching of either cation, if any, was limited. In the zero-gap configuration, the absence of a circulating liquid electrolyte also limits the transport of dissolved species away from the electrode surface, and any Cu^2+^ or Fe^3+^ ions released would be retained by ion exchange at the sulfonic acid groups of the membrane. However, neither the membrane nor the feed water was analyzed for dissolved metals in this study; therefore, a minor leaching, which may contribute to the local loss of adhesion of the film, cannot be excluded. Its quantification by inductively coupled plasma mass spectrometry of the membrane and of the water will be included in future durability studies.

### 3.11. Degradation Pathways and Implications for Device Design

The combination of electrochemical and post-operando analyses provides a consistent link between device performance and materials stability. At the device level, the invariant series resistance, the smooth current decay and the full recovery after each dark interval show that the cell, although assembled without any housing and fed only by wetting the membrane edges, preserved its ionic and electronic continuity for 96 h. The ≈5–6% decrease in the saturated photocurrent is mirrored by an equivalent increase in the photo-induced charge-transfer resistance and of the amplitude of the dominant DRT peak, while the low-bias response and the time scale of the interfacial process are essentially unchanged.

From a mechanistic perspective, WO_3_ undergoes a mild surface reduction (increase in W^5+^ and oxygen-vacancy-related species), hydration and a partial loss of long-range order. In the strongly acidic environment provided by the ionomer, photogenerated electrons that are not promptly extracted, particularly under bias-free conditions, can be charge-compensated by proton insertion, generating W^5+^ centers and disordered domains; in addition, peroxo species formed during water oxidation in acidic media may contribute to surface corrosion [32]. Nevertheless, the retention of crystalline cores, film coverage and tungsten content shows that WO_3_ is structurally robust in the zero-gap acidic configuration, and the ionomer residue on its surface testifies to an intimate photoanode/membrane contact. Conversely, CuFe_2_O_4_ retains its Fe^3+^ framework and bulk stoichiometry, but it undergoes a partial Cu^2+^ → Cu^+^ reduction and hydroxylation at the surface and, above all, a local loss of adhesion to the FTO substrate. Such detachment reduces the photoactive area and, consequently, the saturated photocurrent without affecting the photocurrent onset, which is exactly what is observed in the *J*–*V* curves. Therefore, the CuFe_2_O_4_ photocathode, and in particular its adhesion and surface stability in contact with the acidic ionomer, is identified as the lifetime-limiting component of the device, in analogy with the role of CuO in our alkaline hematite/CuO prototype; however, the spinel ferrite proved considerably more robust than CuO, which underwent bulk electro-reduction to Cu_2_O/Cu after 240 h of operation (Section 3.8).

The relatively high durability of the unprotected CuFe_2_O_4_ photocathode deserves comment, since p-type oxide photocathodes are generally susceptible to photocorrosion, either through the reduction of lattice cations by photogenerated electrons or through their oxidation by photogenerated holes [33,34]. Signs of photoreduction have also been reported for CuFe_2_O_4_ photocathodes in alkaline media [39]. Several factors are expected to contribute to the stability observed in the present device. First, in the spinel lattice, the Fe^3+^–O framework, which was preserved during operation (Section 3.9), is expected to confine the reduction of Cu^2+^ to the near-surface region, whereas in CuO the reduction propagated into the bulk [43]. Second, in the tandem configuration, the photocathode is illuminated only by the photons transmitted by WO_3_ (*λ* > ≈460 nm, Section 3.1) and operates at low current densities (≤0.5 mA cm^−2^), which limits the rate of charge accumulation at its surface. Third, holes are the majority carriers in p-type CuFe_2_O_4_ and are continuously extracted through the FTO back contact, where they recombine with the electrons supplied by the photoanode through the external circuit; this efficient extraction limits the oxidative photocorrosion pathway. Fourth, the absence of a liquid electrolyte and the N_2_ atmosphere surrounding the cell exclude both the attack by atmospheric O_2_ and the continuous removal of dissolution products by a flowing electrolyte, as discussed in Section 3.10. Nevertheless, the partial Cu^2+^ → Cu^+^ reduction and the local delamination show that the photocathode remains the weak point of the device, and protective layers and co-catalysts will be required for operation over thousands of hours.

The different stability of the two photoelectrodes highlights the importance of stabilization strategies for the photocathode. Potential solutions include: (i) the enhancement of the adhesion of CuFe_2_O_4_ through optimized spray and annealing parameters or compact interlayers; (ii) thin protective overlayers, such as TiO_2_ deposited by atomic layer deposition [70,71,72]; (iii) earth-abundant co-catalysts that accelerate the OER at WO_3_ and the HER at CuFe_2_O_4_ and suppress the self-reduction of Cu^2+^ [46]; (iv) the integration of the optimized WO_3_/CuFe_2_O_4_ pair into the modular, scalable zero-gap platforms already developed for larger active areas [44]; and (v) the replacement of water oxidation at the acid-stable WO_3_ photoanode with value-added anodic reactions, such as the photoelectrochemical production of peroxydisulfate in bisulfate-containing anolytes, which can be coupled with solar H_2_ evolution at the photocathode [26].

### 3.12. Comparison with Other Tandem Cells and Practical Considerations

To place the present results in context, Table 4 compares the main figures of merit of the zero-gap WO_3_/CuFe_2_O_4_ cell with those of representative oxide-based tandem PEC cells and of our previous membrane-based devices. In terms of bias-free efficiency, the present device (STH ≈ 0.05%) remains well below the best all-oxide tandems, such as BiVO_4_–Cu_2_O cells, which reached ≈0.5% [20] and ≈3% [73], and below multimediator-modulated cells exceeding 4% [22]. However, these higher efficiencies were obtained in liquid electrolytes at near-neutral or weakly alkaline pH, with co-catalysts (e.g., Co-Pi, RuO_x_ and NiMo) and, for Cu_2_O, with buried junctions and protective overlayers deposited by atomic layer deposition [20,73]; moreover, the photocurrent of the BiVO_4_–Cu_2_O tandem of ref. [20] decayed within minutes because of catalyst detachment. The most directly comparable system is the unprotected, co-catalyst-free WO_3_–CuCrO_2_ tandem operated in 0.1 M HClO_4_ [24]: with respect to that device, the present cell delivers an approximately four-fold higher bias-free current density (≈42 vs. ≈10 µA cm^−2^) and an approximately eight-fold higher STH efficiency (≈0.05% vs. 0.006%), and it operated for 48 h at 0 V (96 h in total) instead of 3 h, without any liquid electrolyte. In addition, unlike our previous membrane-based devices with hematite and CuO photoelectrodes, which were evaluated under an assisting bias [42,43,74], the present cell operates without any external bias and, under bias, in a photo-limited rather than bias-driven regime.

Beyond the absolute efficiency, the merits of the present configuration lie in its materials and architecture. (i) Both photoelectrodes consist of earth-abundant, non-critical elements and are prepared by simple, scalable solution-based routes (electrodeposition and spray coating) without co-catalysts or protective layers, so that the intrinsic performance and degradation of the oxide pair can be assessed without the contribution of additional components. (ii) The spectral complementarity of the pair is very favorable: the band gap of CuFe_2_O_4_ (1.43 eV) is considerably narrower than those of the photocathodes commonly paired with wide-band-gap photoanodes, such as Cu_2_O (≈2.0 eV), CaFe_2_O_4_ (≈1.9 eV) or CuCrO_2_ (≈3.1 eV) [19,24,33], so that the photocathode harvests the visible and near-infrared photons transmitted by WO_3_ up to ≈870 nm, with a combined photoactive capture above 90% over most of the 300–740 nm range (Section 3.1). (iii) The flat-band potential of CuFe_2_O_4_ is more positive than that of WO_3_, which provides the overlap window required for bias-free operation (Section 3.2). (iv) The zero-gap membrane architecture eliminates the liquid electrolyte, keeps ohmic losses negligible (the series resistance of ≈15 Ω cm^2^ corresponds to an ohmic drop below 10 mV at the plateau current), separates the evolved gases and is directly compatible with the components and manufacturing methods of mature proton-exchange membrane electrolyzers and fuel cells [1], which is a significant advantage in view of scale-up [10,44]. (v) WO_3_ is one of the few oxide photoanodes that are stable in strongly acidic media [25,26], which makes the combination of WO_3_ with a proton-exchange membrane a natural choice for acidic operation.

Finally, the choice between acidic (proton-exchange membrane) and alkaline (anion-exchange membrane) tandem cells involves trade-offs that are relevant for practical applications. Acidic devices benefit from the high proton conductivity and the chemical and mechanical robustness of perfluorosulfonic acid membranes, from the fast kinetics of the HER in acidic media and from the maturity of proton-exchange membrane electrolysis, which delivers high-purity hydrogen and allows operation at elevated pressure [1]; moreover, they are not affected by the carbonation issues of alkaline electrolytes [23]. Their main drawbacks are the limited number of photoelectrode materials and earth-abundant catalysts that are stable at low pH, which essentially restricts the choice of oxide photoanodes to WO_3_ and makes the stability of the photocathode critical, as shown in this work, together with the cost of perfluorinated ionomers and the environmental concerns associated with fluorinated polymers. Conversely, alkaline devices based on anion-exchange membranes allow the use of a wider range of earth-abundant oxides, such as hematite and CuO, and of inexpensive Ni- and Fe-based co-catalysts [3,45,46], and our 10 cm^2^ hematite/CuO prototype produced H_2_ with a purity of 99.95% [43]; however, anion-exchange membranes still exhibit lower ionic conductivity and chemical durability than proton-exchange membranes and are sensitive to CO_2_ uptake [3]. In our alkaline devices, the current was mainly bias-driven and the CuO photocathode underwent bulk electro-reduction after 240 h [43], whereas the present acidic WO_3_/CuFe_2_O_4_ device operates in a photo-limited regime and its photocathode degrades mainly through loss of adhesion. Overall, both approaches remain viable: the acidic route offers mature membrane technology and intrinsically separated, high-purity products, whereas the alkaline route offers a broader choice of materials. For acidic tandem cells, the development of acid-stable photocathodes and of low-cost proton conductors will be key to practical deployment.

## 4. Conclusions

This work demonstrates a 1 cm^2^ zero-gap tandem photoelectrochemical cell that relies exclusively on earth-abundant, unprotected and co-catalyst-free oxide photoelectrodes, namely an electrodeposited nanostructured WO_3_ photoanode and a spray-coated nanostructured CuFe_2_O_4_ photocathode, combined with a Nafion 117 proton-exchange membrane acting as solid electrolyte and separator. The cell was assembled without any external housing and operated with deionized water only, showing that a proton-exchange membrane assembly, a well-established component of electrolyzers and fuel cells, can also serve as the core of an all-oxide PEC tandem operating in an acidic environment.

UV–Vis spectroscopy and Tauc analysis confirm band gaps of 2.75 eV (WO_3_) and 1.43 eV (CuFe_2_O_4_), with a combined photoactive capture above 90% over most of the 300–740 nm range, while Mott–Schottky measurements in N_2_-purged 0.1 M HClO_4_ establish the n-type and p-type character of the photoanode and of the photocathode, with flat-band potentials of ≈+0.38 and ≈+0.82 V vs. RHE, respectively. With respect to the three questions posed in the Introduction, the following answers can be given:

(i) The zero-gap WO_3_/Nafion 117/CuFe_2_O_4_ tandem cell can indeed operate without external bias, delivering ≈42 µA cm^−2^ at 0 V (STH ≈ 0.05%, assuming unity Faradaic efficiency) for 48 h with only a minor decrease. Under a moderate assisting bias, the photocurrent saturates at ≈0.5 mA cm^−2^ for cell voltages more negative than ≈−0.7 V, with a maximum throughput efficiency of 0.70% at −1.0 V, while dark currents remain low (≤0.055 mA cm^−2^), indicating that the device operates in a photo-limited regime. Charge-balance considerations show that photocorrosion can account for only a minor fraction of the charge passed, and the bias-free current density is about four times higher than that of the unprotected WO_3_–CuCrO_2_ tandem operated in liquid 0.1 M HClO_4_, for a much longer operation time.
(ii) The energy-level alignment, with the flat-band potential of the photocathode more positive than that of the photoanode, accounts for the bias-free operation. The combination of EIS and DRT reveals that the device response is governed by a millisecond-scale interfacial charge-transfer process (*τ* ≈ 1 ms), whose resistance decreases by ≈91% under illumination at −0.7 and −1.3 V, whereas the ohmic resistance (≈15 Ω cm^2^) is small and invariant, and a minor microsecond process is associated with charge transport across the solid–solid contacts.
(iii) After 96 h of operation, the saturated photocurrent decreases by only ≈5–6%, in agreement with a ≈6% increase in the photo-induced charge-transfer resistance, while the ohmic resistance and the low-bias response are unchanged. Post-operando analyses show that WO_3_ undergoes mild surface reduction (W^5+^), hydration and a partial loss of long-range order, and retains a residual ionomer layer, but preserves its crystalline cores, coverage and composition. CuFe_2_O_4_ retains its Fe^3+^ framework and Cu/Fe stoichiometry, but shows a partial surface Cu^2+^ → Cu^+^ reduction and, above all, a local delamination from the FTO substrate, which identifies the photocathode as the lifetime-limiting component of the device.

Overall, these results demonstrate that unprotected, co-catalyst-free oxide photoelectrodes prepared by simple solution-based routes can be integrated into a compact proton-exchange membrane tandem cell capable of stable bias-free photoelectrochemical operation. Future work will focus on improving the adhesion and the surface stability of the CuFe_2_O_4_ photocathode, on the integration of earth-abundant co-catalysts and protective overlayers, and on the scale-up of the optimized WO_3_/CuFe_2_O_4_ pair to housed zero-gap devices with active areas of at least 10 cm^2^, which will also enable the gas-chromatographic quantification of the Faradaic efficiency, as previously done for our alkaline prototype, and the analysis of possible metal leaching from the photocathode.

## Figures and Tables

**Table 2:** Equivalent-circuit parameters obtained by fitting the EIS spectra of the tandem cell at the end of test (EoT, after the 96 h durability protocol) with the circuit shown in Figure 5a.

Condition	*R*_s_ (Ω cm^2^)	*R*_p1_ (Ω cm^2^)	*Y*_1_ (µS cm^−2^ s*^n^*)	*n*_1_	*R*_p2_ (Ω cm^2^)	*Y*_2_ (µS cm^−2^ s*^n^*)	*n*_2_
0 V, dark	15.3	37.1	0.179	0.94	15,762.9	0.314	0.85
0 V, light	15.1	29.4	0.235	0.95	12,778.1	0.360	0.86
−0.7 V, dark	14.9	32.9	0.201	0.90	14,541.1	0.325	0.85
−0.7 V, light	15.3	21.0	0.329	0.91	1418.9	2.38	0.87
−1.3 V, dark	15.1	30.9	0.215	0.94	14,322.3	0.315	0.85
−1.3 V, light	14.9	18.9	0.366	0.94	1343.1	2.26	0.87

Conditions indicate the cell voltage and whether the spectra were recorded in the dark or under AM 1.5G illumination (100 mW cm^−2^). *Y* and *n* are the parameters of the constant phase elements, *Z*_CPE_ = 1/[*Y*(*jω*)*^n^*] (Equation (3)); 1 µS cm^−2^ s*^n^* = 10^−6^ S cm^−2^ s*^n^*.

**Table 3:** Key performance and impedance descriptors of the tandem cell at the beginning (BoT) and at the end (EoT) of the 96 h durability protocol.

Descriptor	BoT	EoT	Variation
Photocurrent density *j*_p_ at −1.0 V (mA cm^−2^)	−0.474	−0.448	−5.5%
Throughput efficiency *η*_tp_ at −1.0 V (%)	0.699	0.66	−5.6%
*R*_s_, light, −1.3 V (Ω cm^2^)	14.7	14.9	+1.4%
*R*_p1_, light, −1.3 V (Ω cm^2^)	18.0	18.9	+5.0%
*R*_p2_, light, −1.3 V (Ω cm^2^)	1263.3	1343.1	+6.3%
*Y*_2_, light, −1.3 V (µS cm^−2^ s*^n^*)	1.80	2.26	+25.6%
Main DRT peak position *τ*, light, −1.3 V (s)	9.9 × 10^−4^	9.9 × 10^−4^	≈0%
Main DRT peak amplitude *γ*, light, −1.3 V (Ω cm^2^)	≈1.06 × 10^3^	≈1.12 × 10^3^	≈+6%

**Table 4:** Comparison of the zero-gap WO_3_/Nafion 117/CuFe_2_O_4_ tandem cell with representative oxide-based tandem photoelectrochemical cells and with our previous membrane-based devices.

Photoanode/Photocathode (Additional Layers)	Electrolyte and Configuration	Bias-Free and Bias-Assisted Performance	Stability	Ref.
BiVO_4_ (Co-Pi)/Cu_2_O (Al:ZnO, TiO_2_, RuO_x_)	0.5 M Na_2_SO_4_ with phosphate buffer, pH 6; liquid electrolyte	STH ≈ 0.5% (bias-free)	Photocurrent decay within minutes	[20]
BiVO_4_/Cu_2_O nanowires (Ga_2_O_3_, TiO_2_, NiMo)	Weakly alkaline aqueous electrolyte; liquid electrolyte	STH ≈ 3% (bias-free)	Photocathode stable for >100 h	[73]
WO_3_/CuCrO_2_ (none)	0.1 M HClO_4_; two-compartment cell with Nafion separator; 2 cm^2^	≈10 µA cm^−2^, STH 0.006% (bias-free)	>3 h	[24]
Fe_2_O_3_ (NiFeO_x_)/CuO on hydrophobic carbon backing	Zero-gap, anion-exchange membrane	*η*_tp_ ≈ 2% at ≈0.6 V bias	–	[42]
Ti-doped Fe_2_O_3_/CuO	Zero-gap glass electrode membrane assembly; 40 cm^2^	*η*_tp_ up to 1.2% (bias-assisted)	–	[74]
Fe_2_O_3_/CuO	Zero-gap, anion-exchange membrane, housed; 10 cm^2^	Bias-driven regime; H_2_ purity 99.95%	240 h; bulk reduction of CuO	[43]
WO_3_/CuFe_2_O_4_ (none)	Zero-gap, Nafion 117, deionized water feed, no liquid electrolyte; 1 cm^2^	≈42 µA cm^−2^, STH ≈ 0.05% ^a^ (bias-free); *η*_tp_ = 0.70% at −1.0 V	96 h (48 h at 0 V + 48 h at −0.7 V); −5 to −6%	This work

^a^ Calculated assuming unit Faradaic efficiency. STH, solar-to-hydrogen efficiency; *η*_tp_, throughput efficiency (Equation (1)); –, not specified in a comparable form.

## Data Availability

The raw data supporting the conclusions of this article will be made available by the authors on request.

## Supplementary Materials

The following supporting information can be downloaded at https://www.mdpi.com/article/10.3390/nano16211415/s1: Figure S1: Tauc and Mott–Schottky plots of the WO_3_/FTO photoanode and CuFe_2_O_4_/FTO photocathode; Figure S2: *J*–*V* curves, photocurrent density and throughput efficiency of the tandem cell at the end of test; Figure S3: Nyquist plots of the tandem cell at the end of test; Figure S4: XRD patterns of the fresh and used WO_3_/FTO photoanode and CuFe_2_O_4_/FTO photocathode; Figure S5: EDS spectra of the fresh and used photoelectrodes; Figure S6: W 4f and O 1s XPS spectra of the fresh and used WO_3_ photoanode; Figure S7: C 1s, F 1s and S 2p XPS spectra of the fresh and used WO_3_ photoanode; Figure S8: TEM images of the fresh and used photoelectrode materials; Table S1: Characteristic time constants of the equivalent-circuit elements at the beginning and at the end of test and comparison with the DRT peaks.

## Abbreviations

The following abbreviations are used in this manuscript:

AM 1.5G	Air mass 1.5 global solar spectrum
BoT	Beginning of test
CB	Conduction band
CPE	Constant phase element
DRT	Distribution of relaxation times
EDS	Energy-dispersive X-ray spectroscopy
EIS	Electrochemical impedance spectroscopy
EoT	End of test
FE-SEM	Field-emission scanning electron microscopy
FTO	Fluorine-doped tin oxide
HER	Hydrogen evolution reaction
*J*–*V*	Current density–voltage
OER	Oxygen evolution reaction
PDF	Powder Diffraction File
PEC	Photoelectrochemical
RHE	Reversible hydrogen electrode
STH	Solar-to-hydrogen
TEM	Transmission electron microscopy
VB	Valence band
XPS	X-ray photoelectron spectroscopy
XRD	X-ray diffraction
