# Supplementary material for: Zero-Gap Tandem Photoelectrochemical Cell Based on Nanostructured WO3 and CuFe2O4 Photoelectrodes and a Proton-Exchange Membrane: Bias-Free Photoelectrochemical Operation and Degradation Analysis
Source: Nanomaterials (Basel). 2026 Nov 3;16(21):1415. doi: 10.3390/nano16211415 (PMC13764984; doi:10.3390/nano16211415)
Supplement: Supplementary file 1 [file nanomaterials-16-01415-s001.zip › nanomaterials-4662812-supplementary.pdf]

SUPPLEMENTARY INFORMATION

Zero-Gap Tandem Photoelectrochemical Cell Based on Nanostructured WO_3 and $CuFe_2O_4$ Photoelectrodes and a Proton-Exchange Membrane: Bias-Free Photoelectrochemical Operation and Degradation Analysis

Rossana Giaquinta, Carmine Arnese and Stefano Trocino *

Institute for Advanced Energy Technologies "Nicola Giordano" (ITAE), National Research Council of Italy (CNR), Via Salita S. Lucia Sopra Contesse 5, 98126 Messina, Italy; rossanagiaquinta@cnr.it (R.G.); carmine.arnese@cnr.it (C.A.)

* Correspondence: stefano.trocino@cnr.it; Tel.: +39-090-624-270

This document contains Figures S1–S8 and Table S1, which complement the main text. Figures and tables are numbered in order of citation in the main text; equation and figure numbers without the prefix "S" refer to the main text.

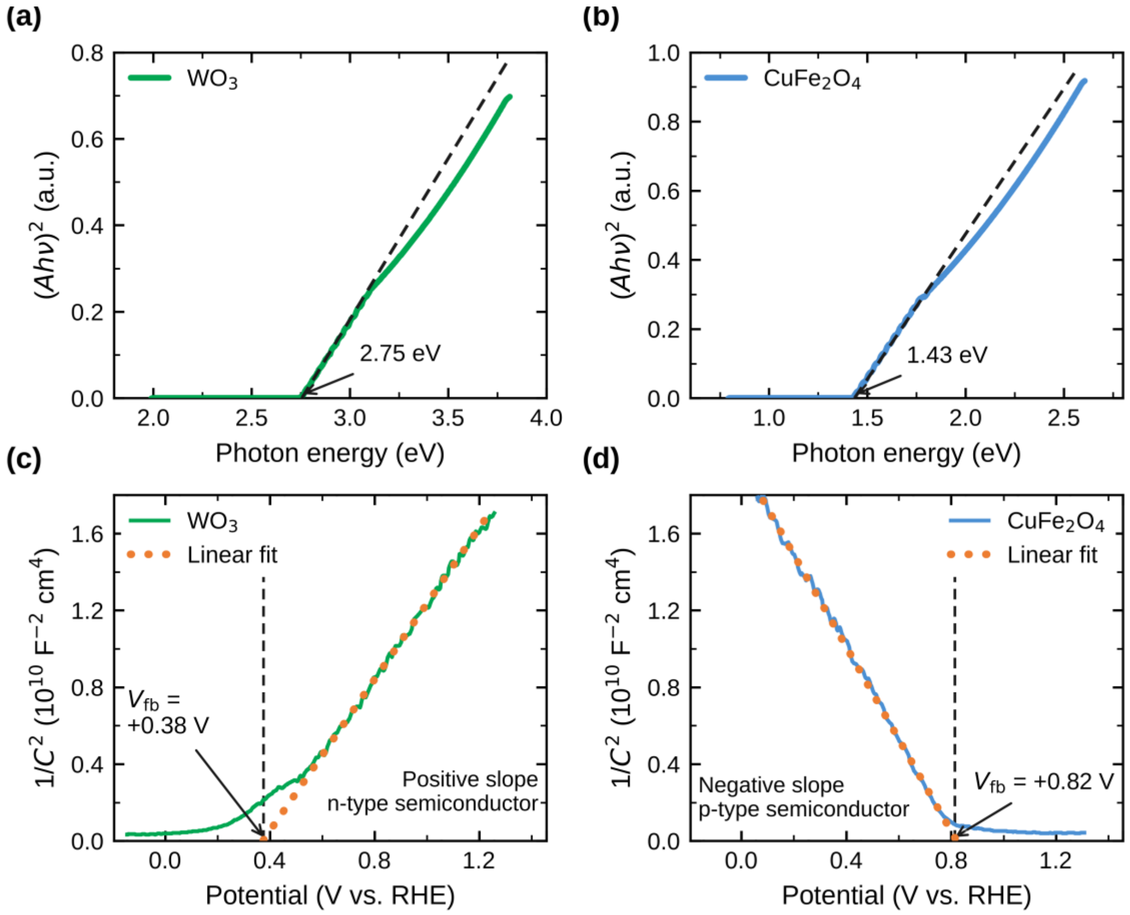

Figure S1. Tauc plots, $(Ah\nu)^2$ vs. photon energy for a direct allowed transition, of (**a**) the WO_3/FTO photoanode and (**b**) the $CuFe_2O_4$/FTO photocathode; dashed lines are the linear extrapolations of the absorption edges, giving optical band gaps of 2.75 and 1.43 eV, respectively. Mott–Schottky plots of (**c**) WO_3/FTO and (**d**) $CuFe_2O_4$/FTO recorded at 1 kHz in N_2-purged 0.1 M $HClO_4$ (pH 1) in the dark (representative of several repeated measurements); the potentials, measured vs. Ag/AgCl (saturated KCl), were converted to the RHE scale with

the Nernst equation (Equation (7) of the main text). Dotted lines are the linear fits, whose intercepts give flat-band potentials (V_{fb}) of +0.38 and +0.82 V vs. RHE (+0.12 and +0.56 V vs. Ag/AgCl), respectively.

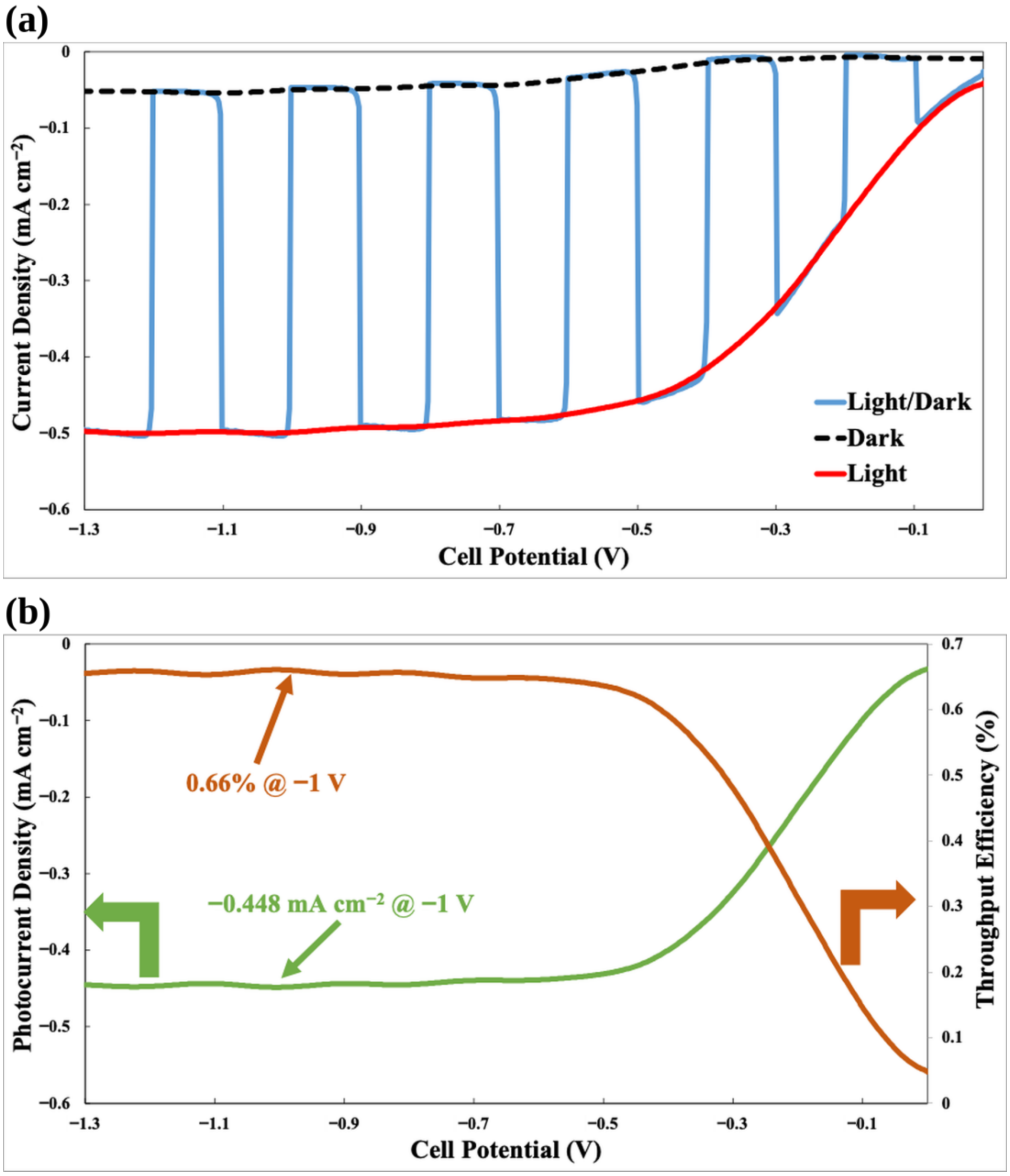

Figure S2. (**a**) Current density–voltage (J–V) curves of the tandem cell at the end of test (EoT), after the 96 h durability protocol, recorded in the dark, under continuous AM 1.5G illumination (100 mW cm^{-2}) and under chopped illumination (5 s light/5 s dark) at a scan rate of 20 mV s^{-1}. (**b**) Photocurrent density ($j_p = j_{light} - j_{dark}$, left axis) and throughput efficiency (η_{tp}, right axis; Equation (1) of the main text) as a function of the cell voltage, derived from the curves in (a).

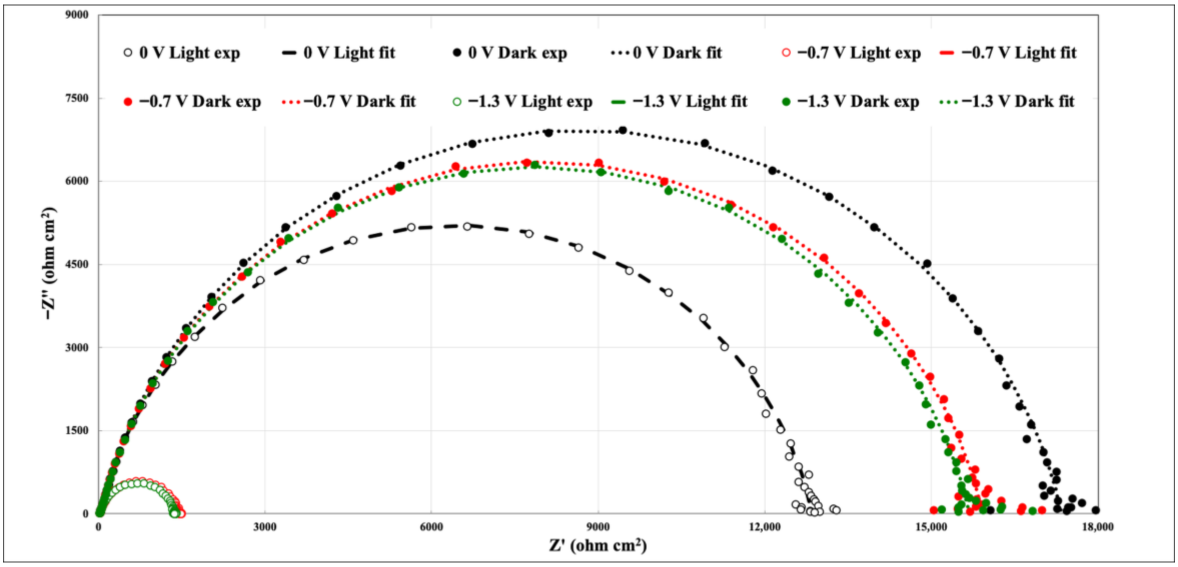

Figure S3. Nyquist plots of the tandem cell at the end of test (EoT), after the 96 h durability protocol, recorded in the dark and under AM 1.5G illumination (100 mW cm^{-2}) at cell voltages of 0, −0.7 and −1.3 V. Symbols represent experimental data and lines represent the fits obtained with the $R_s(R_{p1}\|CPE_1)(R_{p2}\|CPE_2)$ equivalent circuit (Figure 5a of the main text); the fitted parameters are listed in Table 2 of the main text.

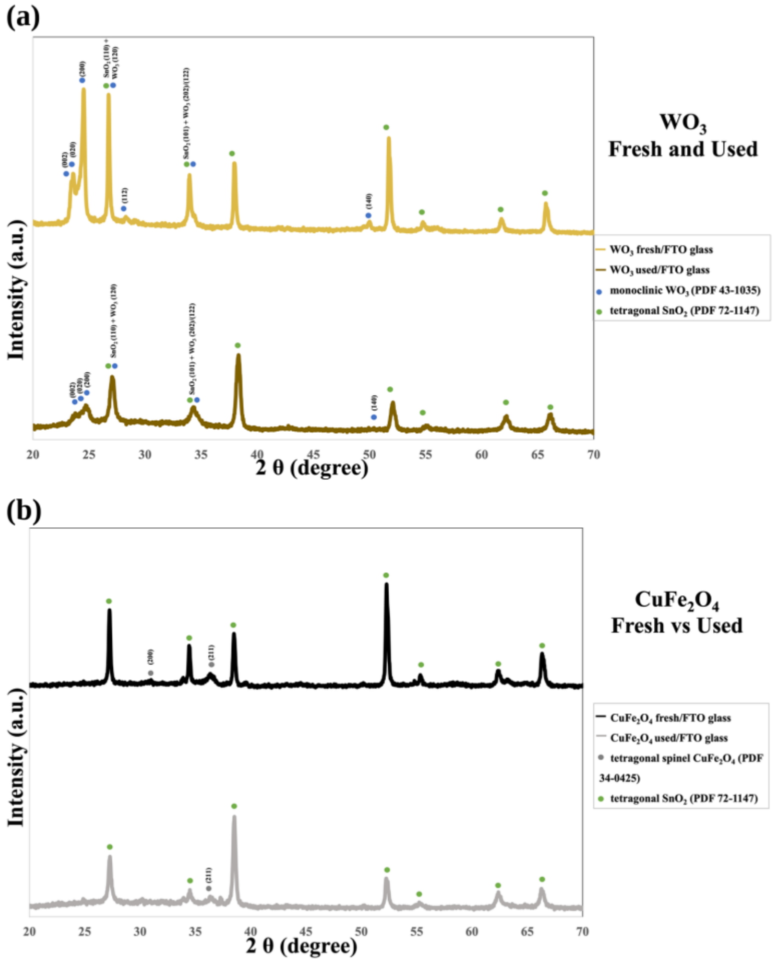

Figure S4. X-ray diffraction (XRD) patterns of (**a**) the WO_3/FTO photoanode and (**b**) the $CuFe_2O_4$/FTO photocathode before electrochemical testing (fresh) and after the 96 h durability test (used). Reflections assigned to monoclinic WO_3 (PDF 43-1035), tetragonal spinel $CuFe_2O_4$ (PDF 34-0425) and tetragonal SnO_2 of the FTO substrate (PDF 72-1147) are indicated.

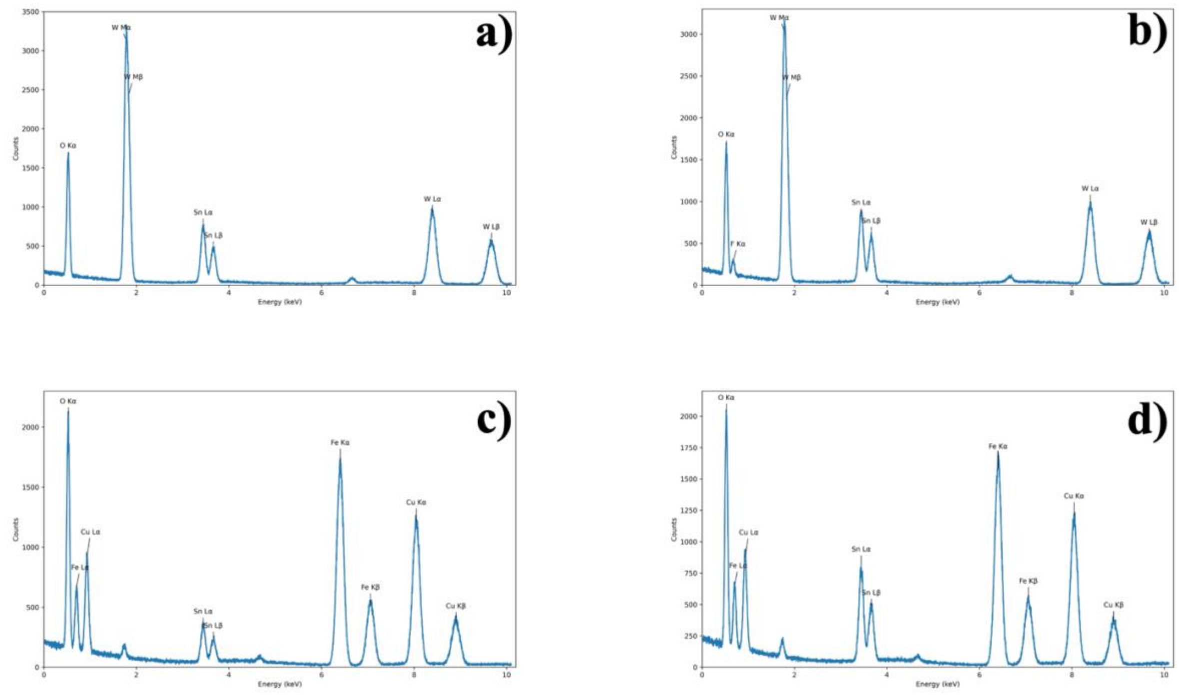

Figure S5. Energy-dispersive X-ray spectroscopy (EDS) spectra of the photoelectrodes before electrochemical testing (fresh) and after the 96 h durability test (used): (**a**) fresh WO_3/FTO; (**b**) used WO_3/FTO; (**c**) fresh $CuFe_2O_4$/FTO; (**d**) used $CuFe_2O_4$/FTO. The labeled peaks correspond to the constituent elements of the oxide films, to the Sn L lines of the FTO substrate and, for the used WO_3, to the F Kα line of the residual Nafion ionomer.

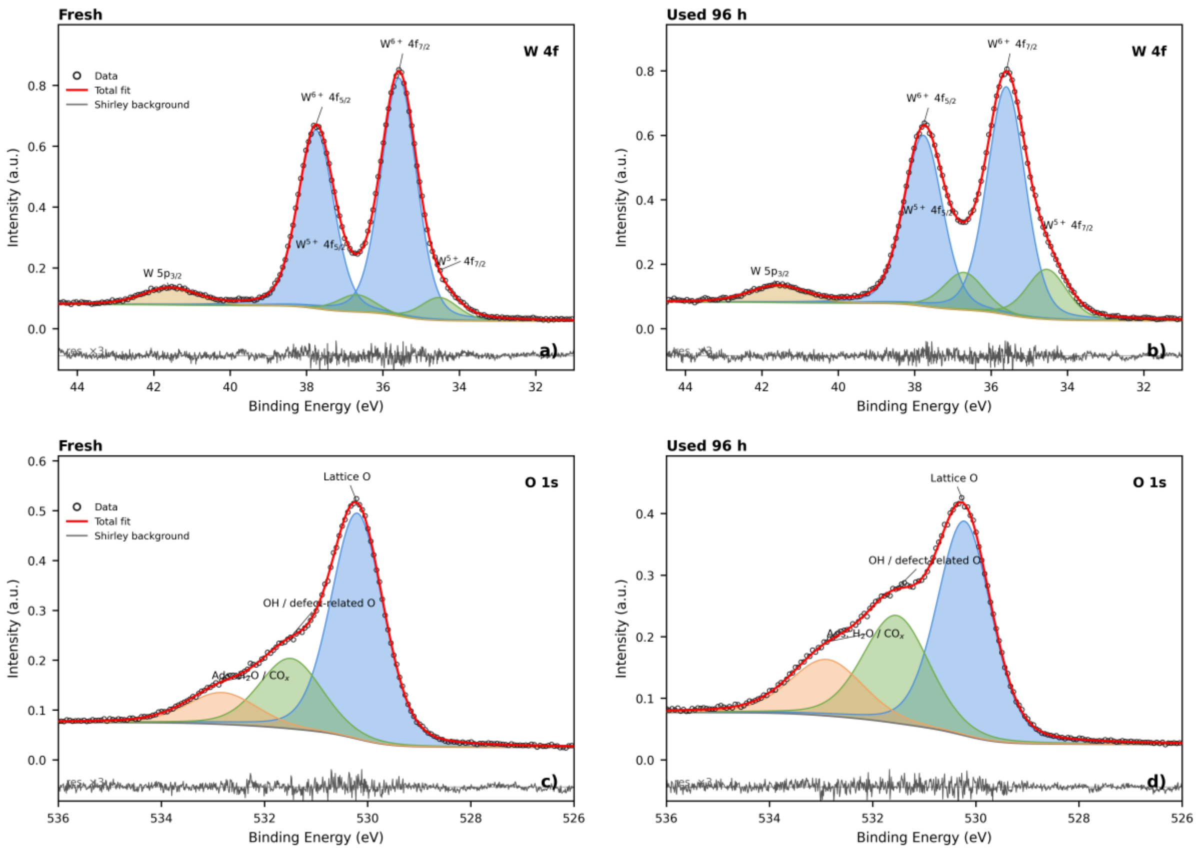

Figure S6. High-resolution XPS spectra and peak deconvolution of the WO_3/FTO photoanode before electrochemical testing (fresh) and after the 96 h durability test (used): (**a**,**b**) W 4f region, including W^{6+} and W^{5+} doublets constrained to a spin–orbit separation of 2.17 eV and a $4f_{5/2}$:$4f_{7/2}$ area ratio of 0.75, together with the W

5p3/2 contribution near 41.6 eV; (**c**,**d**) O 1s region, resolved into lattice oxygen, hydroxyl/oxygen-vacancy-related oxygen (OH/Ov) and higher-binding-energy adsorbed species.

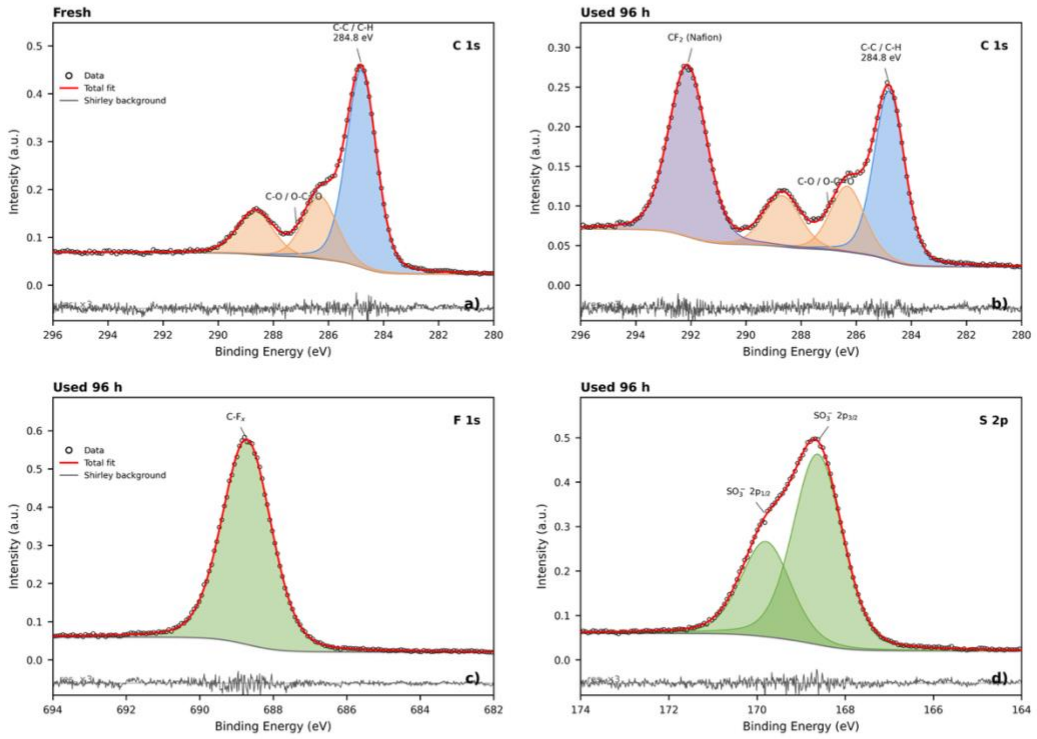

Figure S7. High-resolution XPS spectra and peak deconvolution of the WO_3/FTO photoanode: (**a**) C 1s region of the fresh electrode, resolved into C–C/C–H (284.8 eV, reference), C–O and O–C=O components; (**b**) C 1s region of the used electrode, showing the additional CF_2-related component at 292.1 eV; (**c**) F 1s (C–F_x) and (**d**) S 2p (SO_3^-, $2p_{3/2}$–$2p_{1/2}$ doublet) regions of the used electrode. These signals provide evidence of residual Nafion ionomer at the photoanode surface after operation in the zero-gap cell.

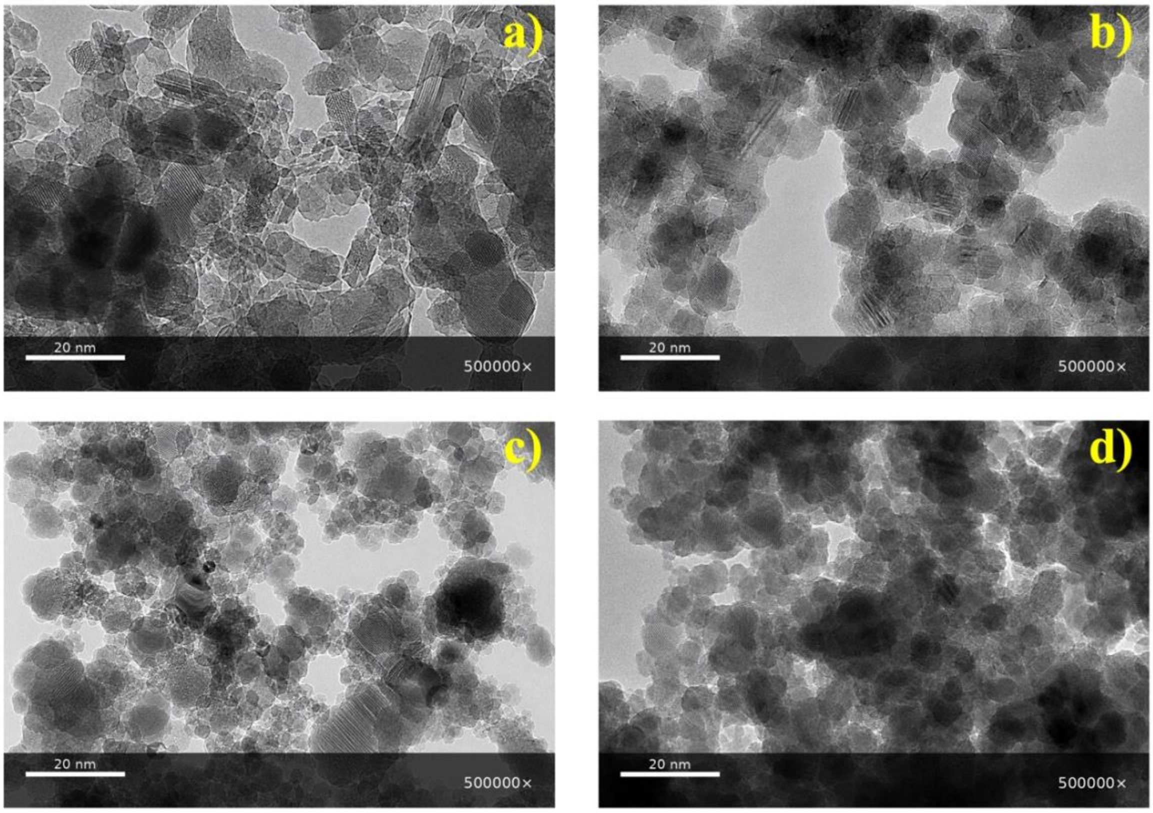

Figure S8. Transmission electron microscopy (TEM) images of the photoelectrode materials before electrochemical testing (fresh) and after the 96 h durability test (used): (**a**) fresh WO_3; (**b**) used WO_3; (**c**) fresh $CuFe_2O_4$; (**d**) used $CuFe_2O_4$. Images were acquired at 500,000× magnification; scale bars are 20 nm.

Table S1. Characteristic time constants of the high-frequency (τ_1) and low-frequency (τ_2) R‖CPE elements of the equivalent circuit, calculated as $\tau = (R{\cdot}Y)^{1/n}$ (Equation (4) of the main text) from the parameters reported in Tables 1 and 2 of the main text, at the beginning (BoT) and at the end (EoT) of the 96 h durability protocol. The positions of the peaks in the distribution of relaxation times (DRT) under illumination at −1.3 V (Figure 8 of the main text) are reported for comparison.

Potential (V)	Condition	τ_1, BoT (μs)	τ_1, EoT (μs)	τ_2, BoT (ms)	τ_2, EoT (ms)
0	Dark	3.17	3.10	1.77	1.94
0	Light	3.63	3.70	1.52	1.92
−0.7	Dark	1.82	1.76	1.68	1.84
−0.7	Light	2.11	2.13	1.14	1.44
−1.3	Dark	2.79	3.10	1.59	1.74
−1.3	Light	0.72	3.24	0.99	1.28
−1.3	Light, DRT peaks	≈0.8	≈3	≈0.99	≈0.99

The DRT peak positions were obtained from the DRT profiles of Figure 8 of the main text; the high-frequency peaks have amplitudes below 50 Ω cm^2.
